# The Arabidopsis SMALL AUXIN UP RNA32 Protein Regulates ABA-Mediated Responses to Drought Stress

**DOI:** 10.3389/fpls.2021.625493

**Published:** 2021-03-12

**Authors:** Yanjun He, Yue Liu, Mengzhuo Li, Anthony Tumbeh Lamin-Samu, Dandan Yang, Xiaolin Yu, Muhammad Izhar, Ibadullah Jan, Muhammad Ali, Gang Lu

**Affiliations:** ^1^Department of Horticulture, College of Agriculture and Biotechnology, Zhejiang University, Hangzhou, China; ^2^College of Agronomy, Northwest A&F University, Yangling, China; ^3^Department of Agriculture, University of Swabi, Swabi, Pakistan; ^4^Key Laboratory of Horticultural Plant Growth, Development and Quality Improvement, Ministry of Agriculture, Zhejiang University, Hangzhou, China

**Keywords:** ABA, Arabidopsis, *AtSAUR32*, drought, PP2C

## Abstract

SMALL AUXIN UP-REGULATED RNAs (SAURs) are recognized as auxin-responsive genes involved in the regulation of abiotic stress adaptive growth. Among the growth-limiting factors, water-deficit condition significantly affects plant growth and development. The putative function of SAUR family member *AtSAUR32* has the potential to diminish the negative impact of drought stress, but the exact function and mode of action remain unclear in Arabidopsis. In the current study, *AtSAUR32* gene was cloned and functionally analyzed. *AtSAUR32* localized to the plasma membrane and nucleus was dominantly expressed in roots and highly induced by abscisic acid and drought treatment at certain time points. The stomatal closure and seed germination of *saur32* were less sensitive to ABA relative to *AtSAUR32*-overexpressed line (OE32-5) and wild type (WT). Moreover, the *saur32* mutant under drought stress showed increased ion leakage while quantum yield of photosystem II (ΦPSII) and endogenous ABA accumulation were reduced, along with the expression pattern of ABA/stress-responsive genes compared with WT and the OE32-5 transgenic line. Additionally, yeast two-hybrid (Y2H) and bimolecular fluorescence complementation (BiFC) assays showed that AtSAUR32 interacted with clade-A PP2C proteins (AtHAI1 and AtAIP1) to regulate ABA sensitivity in Arabidopsis. Taken together, these results indicate that *AtSAUR32* plays an important role in drought stress adaptation via mediating ABA signal transduction.

## Introduction

Being sessile in nature, plants adopt sophisticated mechanism to overcome unavoidable harsh environmental challenges such as extreme temperature and drought, which adversely affect plant growth, development, and productivity. Among these mechanisms are regulation of various metabolic pathways, cellular processes, and activation of stress-resistant genes. Such genes that participate in stress responses can be used to boost crop stress tolerance (Liu et al., 2020). During the period of adaptation, several plant hormones play pivotal functions in stimulating or inhibiting plant development, growth, and stress responses. Among all phytohormones, abscisic acid (ABA) is well known as a stress plant hormone that regulates various molecular and cellular processes including stoma aperture (to control transpiration rate), and transcript levels of stress-responsive genes throughout development (Kuromori et al., 2018; Yoshida and Fernie, 2018) in response to osmotic and drought stress conditions. As an endogenous signal for growth, ABA plays a vital function in the germination of seeds, seedling growth, and development under normal conditions (Humplík et al., 2017). Additionally, when plants experience water deficit, ABA biosynthesis occurs rapidly, moves from the roots to shoots, regulates leaf growth, and induces stomatal closure to counter the stress (Guo et al., 2020).

Furthermore, ABA-dependent and -independent pathways play significant roles in drought stress responses (Shinozaki and Yamaguchi-Shinozaki, 2007). In the ABA-dependent pathway, ABA accumulates during stresses and pyrabactin resistance/pyrabactin resistance-like/regulatory components of ABA receptors (PYR/PYL/RCAR) bind to ABA and clade-A phosphatase type 2C (PP2C.A) to form a PP2C-ABA-PYL ternary complex (Cutler et al., 2010). Binding of ABA-bound PYR/PYL/RCAR to PP2C inhibits the phosphatase activity of PP2C thereby activating SUCROSE NONFERMENTING-1-related protein subfamily 2 kinases (SnRK2s) to phosphorylate ABF/AREB/ABI5 family transcription factors and activates ABA-induced gene expression (Fujita et al., 2009), and ultimately stomatal closure is promoted to overcome the stress (Cutler et al., 2010). Members of clade-A PP2C include *ABI1* (ABA INSENSITIVE 1), *ABI2*, *HAB1* (HYPERSENSITIVE to ABA1), *HAB2*, *AHG1* (ABA HYPERSENSITIVE GERMINATION1), *HAI1* (HIGHLY ABA-INDUCED 1), *HAI2*, and *AIP1* (AKT1-Interacting Phosphatase1) (Hirayama and Shinozaki, 2007). It has been proved that these PP2C genes are generally negative regulators of ABA signaling pathways, which are involved in many ABA-regulated responses, such as rhizogenesis, seed germination, stomatal closure to reduce transpiration rate, inhibition of vegetative growth, and drought-induced resistance (Kim et al., 2013). ABI1 and ABI2 have been verified as negative regulators of ABA signaling and drought stress response. The null mutants of PP2C.A and HAB1 increases ABA response and drought tolerance (Lim et al., 2014, 2015). *hai1* mutant exhibits increased plant sensitivity to ABA signaling, lowers water loss, and results in increased drought tolerance compared to WT (Zhang et al., 2013). Members of the PP2C.A subfamily in rice have also been proposed to mediate drought resistance (Singh et al., 2010). Therefore, PP2C.A members are widely believed to serve very important roles in drought stress responses.

Auxin is vital for regulating plant growth, development, and adaptation to fluctuating environment. However, SMALL AUXIN UP RNAs (SAURs) are primary auxin-responsive genes that are critically involved in auxin signaling pathway and are quickly induced by auxin treatment. Previous studies revealed that SAURs regulate plant developmental and physiological processes (Wu et al., 2012). In Arabidopsis, overexpression of SAUR41 and stabilized fusion proteins of SAUR19 and SAUR63 promote hypocotyl elongation and leaf growth (Chae et al., 2012; Spartz et al., 2012; Kong et al., 2013). Studies have proved that SAUR19 in Arabidopsis can inhibit PP2C.D phosphatases to regulate plasma membrane H^+^-ATPases to promote cell expansion (Spartz et al., 2014). *AtSAUR36* was reported to negatively regulate cell expansion and inhibit leaf growth (Hou et al., 2013). *AtSAUR76* confers reduction in leaf size, but may positively regulate root growth (Stamm and Kumar, 2013). Overexpression of *AtSAUR36* promotes leaf senescence (Hou et al., 2013). Meanwhile, some SAUR genes were proved to participate in tropic growth, apical hook development, seed germination, shade avoidance responses, calcium signaling, and so on (Ren and Gray, 2015). However, functional studies of SAURs were limited in some subgroups and there are few reports on the roles of SAUR in stress responses. Under high temperature, *SAUR19* gene functions downstream of PIF4 to regulate hypocotyl growth (Franklin et al., 2011) and *Pro35S*:GFP-*AtSAUR19* expression confers drought hypersensitivity in tomato (Spartz et al., 2017). The expression levels of many SAUR genes were reduced in response to stress-related hormones: jasmonate and ABA, as well as drought and osmotic stresses (Kodaira et al., 2011). These results indicate that SAUR genes play multiple functions in plant development and defense responses.

Therefore, we conducted this research to functionally characterize a novel gene (*AtSAUR32*) in Arabidopsis plant. We investigated the role of *AtSAUR32* in ABA signaling and drought tolerance through generating *saur32* mutants and overexpressed lines via transgenic approach. Further, AtSAUR32 was verified to interact with HAI1 and AIP1 proteins since their expression levels were increased in *saur32* mutants, while yeast two-hybrid (Y2H) and bimolecular fluorescence complementation (BiFC) assays were used for further confirmation. Besides, we investigated the physiological responses and changes triggered by drought stress in SOD, proline, H_2_O_2_ accumulation, and ion leakage. Together, these findings provide a solid background to understand the vital role of *AtSAUR32* gene in drought stress tolerance.

## Materials and Methods

### Web-Based Analysis of AtSAUR32

For the identification of *cis*-regulatory elements, 1.5 k bp upstream from the start codon (ATG) of the Small auxin up-regulated RNA (SAUR) gene, *AtSAUR32*, was obtained from the Arabidopsis information resource (TAIR)^1^ and queried against the PlantCARE^2^ (Lescot, 2002) (Supplementary Table 1).

### *saur32* Mutants and Transgene Constructs

*Arabidopsis thaliana* plants used in this study were of the Columbia-0 ecotype (Col-0) background. One T-DNA insertion mutant *saur32* (*SALK_033535*) was obtained from the Arabidopsis Biological Resource Center, which has been previously reported to lack *AtSAUR32* expression (Park et al., 2007). In order to identify the homozygous T-DNA insertion, the genomic DNA of mutant seedlings were submitted to PCR genotyping using the primer list in Supplementary Table 2. The seeds were surface-sterilized, stratified at 4°C for 3 days, and germinated on 1/2 MS medium for 10 days. Seedlings were then transferred to a growth chamber at 22/18°C cycle with a photoperiod of 12/12 h (light/dark) under 60% relative humidity for further growth.

To construct vectors for *AtSAUR32* overexpression, the coding region was cloned into the PB7YWG2.0 Gateway vector (Invitrogen) fused with an enhanced yellow fluorescent protein (YFP) tag and driven by the constitutive 35S promoter (primer pairs listed in Supplementary Table 3). Then, the recombinant plasmids were transformed into *Agrobacterium tumefaciens* strain GV3101. *Agrobacterium*-mediated transformation was performed using wild-type (WT) Arabidopsis by floral dip method (Zhang et al., 2006; Ali et al., 2020b). Transgenic plants were selected and then screened with 0.1% Basta for their ability to overexpress *AtSAUR32*. The T_4_ homozygous transgenic plants were used for all experiments.

### Y2H and BiFC Assay

The full-length coding sequence of *AtSAUR32* was amplified by PCR for Y2H assay, using the primer pairs listed in Supplementary Table 4. *AtSAUR32* sequence was ligated into the pGBKT7 vector by restriction site *EcoR* I and *Sma* I, while *HAI1* and *AIP1* were cloned into pGADT7 with the same enzyme sites to form pGADT7-*HAI1*/*AIP1* vectors as prey vector. Two pairs of vectors (pGBKT7-*AtSAUR32*+pGADT7-*HAI1*, pGBKT7-*AtSAUR32*+pGADT7-*AIP1*) and control vectors (pGBKT7-53+pGADT7-T1, pGBKT7-Lam+pGADT7-T1) were transformed into yeast strain AH109 according to the manufacturer’s protocol (Clontech, Beijing, China). The transformed yeast was selected on SD/-Leu-Trp-His-Ade dropout medium. The colonies that contained two interacting proteins grew up after incubation at 30°C for 3 days.

For the BiFC assay, the coding sequence of *AtSAUR32* was fused with N-YFP to generate N-terminal in-frame fusions with N-YFP, and CDS of *HAI1* and *AIP1* were fused with C-YFP to generate C-terminal in-frame fusions with C-YFP as described earlier (Cui et al., 2007). The plasmids were introduced into *A. tumefaciens* (C58C1) as verified through sequencing, and infiltrated into *Nicotiana benthamiana*. After 48 h of co-infiltration, the tissues were analyzed. The fluorescence signal was visualized using a Zeiss LSM710 confocal microscope, and images were superimposed using ZEISS LSM710 software.

### Protein Localization Assay

The coding sequence of AtSAUR32 was amplified and cloned into pFGC-eGFP vector by using the standard molecular techniques (primers, Supplementary Table 5) (Ouyang et al., 2016). The transient expression of AtSAUR32 tagged with GFP was achieved in tobacco epidermal cells. In detail, the construct was transferred into GV3101-competent cells followed by harvesting cells and then added 200 μM acetosyringone, 10 mM MES (pH 5.5), and 10 mM MgCl_2_ and subsequently injected into 4-week-old tobacco seedlings through the leaves using needless syringe. The tobacco plants were kept in the dark for 48 h and then shifted to growth chamber for further 2–3 days. The tobacco leaf cells were visualized using a fluorescent confocal microscope (OLYMPUS BX63, Tokyo, Japan) with an emission of 509 nm and a 488-nm excitation wavelength.

### Measurement of Ion Leakage and Water Loss

The ion leakage rate was measured as described by Liu et al. (2017) with a little modification (Liu et al., 2017). Leaf samples were submerged in 10 ml of distilled water followed by incubation in boiling water (100°C) for 20 min; the conductivities C_1_ (initial before heating) and C_2_ (after cooling) were measured using a conductivity meter (Model DDS-11A, Shanghai Leici Instrument Inc., Shanghai, China). The ion leakage was calculated as (C_1_/C_2_) × 100.

The water loss assay was assessed in detached leaves of 4-week-old *AtSAUR32*-overexpressed, *saur32*, and WT plants placed in a growth chamber with 40% relative humidity. The fresh weight (FW) was recorded immediately after the designated interval of time [0, 0.5, 1, 2, 4, 8, and 12 h post-treatment (hpt)]. The proportion of water lost was calculated as described by Yoo et al. (2010), and the experiment comprised three replicates (Yoo et al., 2010).

$$\mathrm{Water}\,\mathrm{loss}\,\mathrm{rate}\,\frac{\mathrm{Initial}\,\mathrm{FW}-\mathrm{Final}\,\mathrm{FW}}{\mathrm{Initial}\,\mathrm{FW}}\times 100$$

### ROS Detection by Histochemical Staining

The accumulation of hydrogen peroxide (H_2_O_2_) was performed as previously described (Ali et al., 2020a). The fully expanded leaves from the top of control and drought-stressed WT, *ATSAUR32*-overexpressed, and *saur32* mutant plants were detached and accumulation of H_2_O_2_ in leaves was detected by staining with diaminobenzidine (DAB) in 50 mM *Tris*-acetate (pH 3.8) after incubation at 25°C in the dark for 8 h. Chlorophyll was removed by immersing in 80% ethanol, at 70°C for 10 min and stained images were photographed.

### Seed Germination Experiment

The seeds of WT and *saur32* were surface-sterilized, stratified at 4°C for 3 days, and then grown on 1/2 MS medium (0 and 1 μM ABA); seed germination rates were measured at 12-h intervals (0, 12, 24, 36, and 48 h), and seed germination percentage was calculated using the formula below. Further, to observe seedlings in response to ABA, the WT and *saur32* seeds were germinated on 1/2 MS medium supplemented with 0, 0.2, 0.4, and 0.8 μM ABA. After 10 days, seedlings with expanded cotyledon were counted to calculate the green cotyledons. Each test was performed in more than three biological repeats.

$$\mathrm{Germination}\,\mathrm{percentage}\,\frac{\mathrm{Number}\,\mathrm{of}\,\mathrm{germinated}\,\mathrm{seeds}}{\mathrm{Total}\,\mathrm{number}\,\mathrm{of}\,\mathrm{platted}\,\mathrm{seeds}}\times 100$$

### Stomatal Aperture Conductance

Stomatal aperture of epidermal peels was assessed according to Zhang et al. (2012) with little modifications. Leaves from 4-week-old plants were removed and epidermal peels were incubated in buffer containing a solution of 50 mM KCl, 0.2 mM CaCl_2_, and 10 mM 2-(N-morpholino)-ethane-sulfonic acid (MES)-KOH (pH 6.15) at 22°C under white light (150 pmol m^–2^ s^–1^) for 2 h to open the stomata. The leaves were then transferred to MES-KCl buffer containing 10 μM ABA for 1 and 3 h. During analysis, light was reduced so that it does not affect the stomata size. The length and width of stomatal pore were measured using ImageJ tool (Schneider et al., 2012) and used to calculate the stomatal aperture. The experiment was conducted with three biological replicates.

### Phytohormone Extraction and Quantification

The extraction of the endogenous hormones: abscisic acid (ABA), jasmonic acid (JA), and Auxin (IAA) from *saur32* and WT plants were done according to the described methods with little modifications (Fu et al., 2012; Pan et al., 2019). Plant samples (0.1 g) were ground in liquid nitrogen and homogenized in 1 ml ethyl-acetate having 25 μl solution of standard d6–ABA (OlchemIm Ltd., Czechoslovakia), d5-JA (QCC), and d2-IAA (Sigma-Aldrich). The samples were vortexed, agitated at 140 rpm at 4°C for 12 h, and centrifuged at 12,000 rpm for 10 min, and extraction was repeated with agitation for 1 h. The supernatants were collected and evaporated to dryness using nitrogen gas. 0.5 ml of 70% methanol was added to the dried samples, vortexed, and centrifuged at 12,000 rpm for 10 min at 4°C. 0.2 ml of sample supernatants was placed in snap-cap vials and analyzed using Agilent 1290 infinity HPLC system coupled with Agilent 6460 Triple Quad liquid chromatography-mass spectrometry device (Agilent Technologies, Germany). The Agilent Zorbax XDB C 18 column (150 × 2.1 mm, 3.5 μm) was used for HPLC analysis as described in the previous study (Chen et al., 2018).

### RNA Isolation and Library Preparation

Total RNA was isolated from *saur32* and WT plants (4 weeks old) using TRIzol reagent (Invitrogen, Germany) according to the manufacturer’s instructions. RNA quality was checked by 1% agarose gel electrophoresis, and the concentration was determined by Nanodrop 1000 spectrophotometer (Thermo Scientific Inc.). The RNA samples (two replicates) were sent to Biomarker Company (Beijing, China) for reverse transcription to cDNA and then sequenced on an Illumina HiSeq^TM^ 2000 platform (Jiang et al., 2011). Analysis and identification of differentially expressed genes (DEGs) (log_2_| Fold Change| ≥ 1 and FDR < 0.01) and Gene Ontology (GO) functional and pathway enrichment analyses of DEGs were also carried out by the same company. Raw sequence reads were uploaded into the NCBI Sequence Read Archive with accession number PRJNA688697.

### RNA Extraction and Quantitative Real-Time PCR

Total RNA was extracted from all the samples using TRIzol reagent (Invitrogen, Germany), according to the manufacturer’s instructions. The RNA extraction procedure and cDNA synthesis followed the same procedures as detailed in our recently published papers (Ali et al., 2018, 2020a). For qRT-PCR, specific primer sets were designed and checked for primer specificity in Arabidopsis genome (Supplementary Table 6). qRT-PCR was performed in CFX96 Real-Time System machine (Bio-RAD, Hercules, CA, United States) using SYBR Premix Ex Taq^TM^ II (TaKaRa). Relative expression levels were normalized by Arabidopsis *ACTIN7* gene and calculated using the 2^–ΔΔ*Ct*^ method (Schmittgen and Livak, 2008). Data were presented as means and +SD of three replications obtained from three independent biological experiments.

### Drought Stress and ABA Treatment

For drought treatment, 4-week-old well-watered Arabidopsis *saur32* mutant, *AtSAUR32*-overexpressed, and WT plants were used. The plants were deprived of water for 15 days in the greenhouse. Three biological replicate experiments were conducted for each line. For ABA treatment, 4-week-old Arabidopsis plants were sprayed with 10 μM ABA and control plants were separately sprayed with mock solution and maintained under the same growth environment.

### Determinations of Proline and SOD Content

Free proline content was quantified following the procedure described by Bates et al. (1973). Leaf sample (0.5 g) was homogenized in 10 ml of 3% aqueous sulfosalicylic acid using mortar and pestle, and the homogenate was filtered through Whatman filter paper no. 2. Ninhydrin solution (2.5%) was prepared in glacial acetic acid with further addition of 6 M phosphoric acid. The reaction mixture containing the plant extract (2 ml), glacial acetic acid (2 ml), and ninhydrin fresh solution (2 ml) was mixed and boiled at 100°C for 1 h in a water bath. Toluene (4 ml) was added in cooled reaction mixture tubes and thoroughly shaken. The optical density (OD) values were measured at 520 nm and the proline content (μmol g^–1^ FW) was calculated from standard curve.

Superoxide dismutase (SOD) activity was measured by photoreduction of NBT (Nitro-Blue Tetrazolium). 0.5 g of fresh leaf was ground in mortar with 5 ml of PBS (50 mM PBS, 25 mM NBT, 0.003 mM Riboflavin, and 0.1 mM EDTA, pH 7.8). The extract was centrifuged for 15 min under 4°C at 12,000 rpm. The supernatant was illuminated at 4000 lux for 20 min. SOD activity was spectrophotometrically quantified at 560 nm. Control was measured in the dark according to the abovementioned procedure while the SOD activity was calculated by the method of Dionisio-Sese and Tobita (Stewart and Bewley, 1980).

### Statistical Analysis and Data Plotting

Each experiment was conducted in replicates for authenticity, and data were analyzed by IBM SPSS Statistics 25 while Duncan Multiple Range (DMR) test was applied. The plotted data are the means of three independent experiments and the standard deviation (±SD). Asterisk symbols (^∗^*p* < 0.05; ^∗∗^*p* < 0.01; ^∗∗∗^*p* < 0.001) indicate the significant difference in comparison to WT. Graphs were drawn by GraphPad Prism 8.0 (GraphPad Software, Inc., La Jolla, CA, United States), and PhotoScape X (Pro version 4.0) was used for designing.

## Results

### *In silico* and Expression Analysis of *AtSAUR32*

**FIGURE 1:**
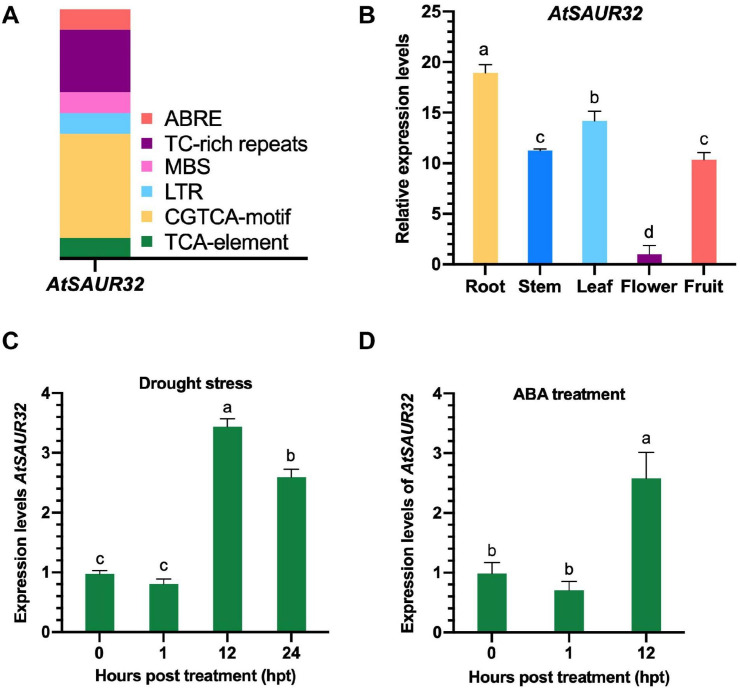
Transcriptomic and qRT-PCR analysis of *AtSAUR32* gene. **(A)** *Cis*-regulatory elements involved in the stimulation of *AtSAUR32* used 1.5 k upstream region and analyzed by the online tool PlantCARE. **(B)** The tissue-specific expression levels of *AtSAUR32* (Arabidopsis plants were grown under normal condition) were examined using qRT-PCR. **(C)** The expression pattern of *AtSAUR32* under drought treatment performed by qRT-PCR analysis. **(D)** The expression pattern of *AtSAUR32* under ABA treatment, examined by qRT-PCR. Mean values and standard deviation (+SD) are plotted as analyzed by Duncan’s multiple range (DMR) test (*P* < 0.05); lowercase letters (a–d) indicate significant difference.

*In silico* analysis of *AtSAUR32* indicated that *cis*-elements conferring response to plant hormones and abiotic stresses were found in the promoter region. Noticeably, the *AtSAUR32* promoter region carried ABA responsiveness elements (ABRE), MeJA-responsiveness elements (CGTCA-motif), and salicylic acid responsiveness elements (TCA-element) shown in Figure 1A and Supplementary Table 7. Besides phytohormone responsive element, some abiotic stress responsive regulatory elements were also detected, such as MYB binding site involved in drought-inducibility (MBS), LTR (low-temperature responsiveness), and TC-rich repeats (defense and stress responsiveness). All of the anticipated *cis*-elements were involved in responses to signaling molecules and stresses.

The qRT-PCR analysis revealed that *AtSAUR32* transcript abundance was evaluated in vegetative (root, stem, and leaf) and reproductive (flower and fruit) tissues in WT Arabidopsis plants. The results showed dominant expression in root (19-fold) followed by leaf (14-fold), while the lowest expression, 1-fold, was recorded in the flower (Figure 1B), implying significant contribution of *AtSAUR32* during the seedling stage of Arabidopsis. For further insight into the transcriptomic features of *AtSAUR32* in Arabidopsis leaves and roots under drought conditions, we performed *in silico* analysis from publicly accessible transcriptomic database of Arabidopsis (Berardini et al., 2015). The diagrams showed the index of expression level ranging from yellow (0) to red (570) (Supplementary Figure 1). The qRT-PCR results indicated that the expression level of *AtSAUR32* in Arabidopsis leaves exhibited higher expression levels at certain time points under drought stress conditions. As demonstrated in Figure 1C, *AtSAUR32* expression was initially at a basal and non-significant level in the first 1 h of drought stress and abruptly increased at 12 hpt to a higher peak (>3.6-fold) and then slightly reduced after 24 h. Overall, *in silico* and qRT-PCR analyses revealed that *AtSAUR32* showed remarkable response to drought at the transcriptional level.

In order to examine whether ABA regulates *SAUR32*, we retrieved publicly available data from Arabidopsis database (Berardini et al., 2015) and confirmed through qRT-PCR analysis. The results revealed high response of *AtSAUR32* to ABA treatment at 3 hpt. The diagrams display the index of expression levels ranging from yellow (0) to red (332) (Supplementary Figure 2). Furthermore, qRT-PCR analysis exhibited that after 12 h, the expression level of *AtSAUR32* was significantly higher compared with 0 and 1 hpt (Figure 1D).

### AtSAUR32 Protein Localized in Cell Membrane and Nucleus

The pFGC-eGFP expression vector containing 35S promoter and green fluorescence protein (GFP) reporter gene was recombined with the ORF portion of AtSAUR32. The *N. benthamiana* plants that expressed as red nuclear marker RFP-H2B protein were infiltrated with pFGC-eGFP and pFGC-eGFP-AtSAUR32 fused plasmids for AtSAUR32 expression in ephemeral tissue. The confocal laser micrographs showed that 35S::*AtSAUR*32::GFP fused protein was mostly localized in the cell membrane and nucleus of the cell (Supplementary Figure 3).

### Effect of Drought on *AtSAUR32* Knockout Arabidopsis Plants

**FIGURE 2:**
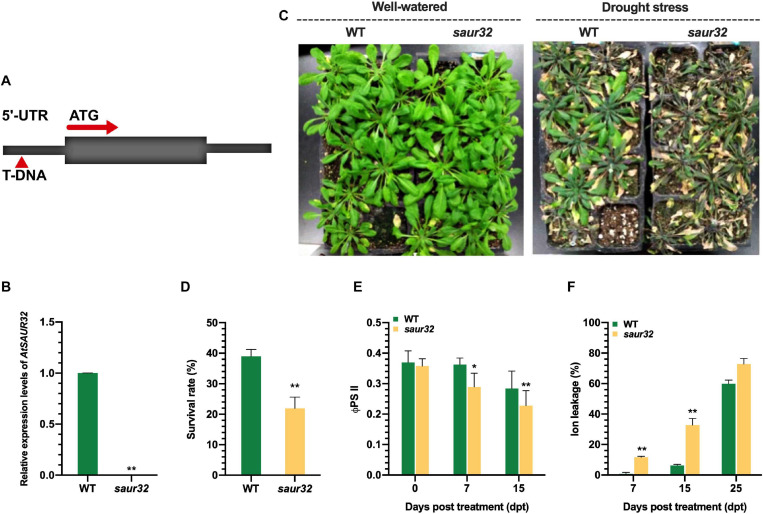
*AtSAUR32* knockout mutant increased sensitivity to drought stress. **(A)** Schematic diagram of T-DNA insertion in *AtSAUR32*. **(B)** Transcript level of *AtSAUR32* in *saur32* mutant and WT plants. **(C)** Phenotypes, well-watered and drought stress recovery assay of *saur32* mutant and WT plants 7 days after rewatering. **(D)** Survival percentage of WT and *saur32* mutant plants 7 days after rewatering. **(E,F)** Effect of drought stress on quantum yield of photosystem II electron transport (ΦPSII) and ion leakage of *saur32* mutant and WT plants, respectively. Mean values and standard deviation (+SD) were plotted as analyzed by Duncan’s multiple range (DMR) test; asterisk (*p < 0.05; **p < 0.01) indicates significant difference.

To explore the protective role of *AtSAUR32* in Arabidopsis against water-deficit conditions, we examined the effects of *AtSAUR32* gene loss of function in knockout mutant. The T-DNA insertion mutant of *AtSAUR32* was determined in SALK collection (Columbia background), corresponding to the donor stock accession SALK_033535, which was named *saur32*. The sequencing of the T-DNA flanking region in *saur32* showed that the insertion was localized on the 5′ UTR region (Figure 2A). Furthermore, we confirmed the *saur32* mutant through qRT-PCR exhibited lack of *AtSAUR32* expression in *saur32* (Figure 2B) in line with Park et al. (2007). Hence, the *saur32* mutant and WT Arabidopsis plants were used for further study. The phenotypes shown in Figure 2C revealed that after rewatering, more WT than *saur32* plants recovered from the drought stress with survival percentage of 39 and 21, respectively (Figure 2D), indicating that *AtSAUR32* loss of function significantly increases the susceptibility of Arabidopsis plants to drought stress. Moreover, the quantum yield of photosystem II electron transport (ΦPSII) and ion leakage were examined in untreated healthy and drought-treated plants. Under drought conditions, the value of ΦPSII was significantly decreased (0.28) in *saur32* at 7 days post-treatment (dpt) as compared with WT (0.39), while it was further decreased with increased time interval (15 dpt) in *saur32* and WT plants (Figure 2E). On the other hand, ion leakage in mutant (*saur32*) plants increased after drought stress, and a highly significant leakage was observed at 7 and 15 dpi in *saur32* plants, which were > 10 and 26% higher than in WT plants, respectively (Figure 2F). However, excessive ion leakage > 72% was recorded at 25 dpt. The determination of ion leakage was an indirect assessment of plasma membrane injury caused by drought stress. These results suggest the prominent role of *AtSAUR32* against drought as the *saur32* knockout mutant lost the ability to survive longer and less defensive in nature against abiotic stresses.

### *AtSAUR32*-Overexpressed Arabidopsis Plants Exhibited Enhanced Drought Tolerance

**FIGURE 3:**
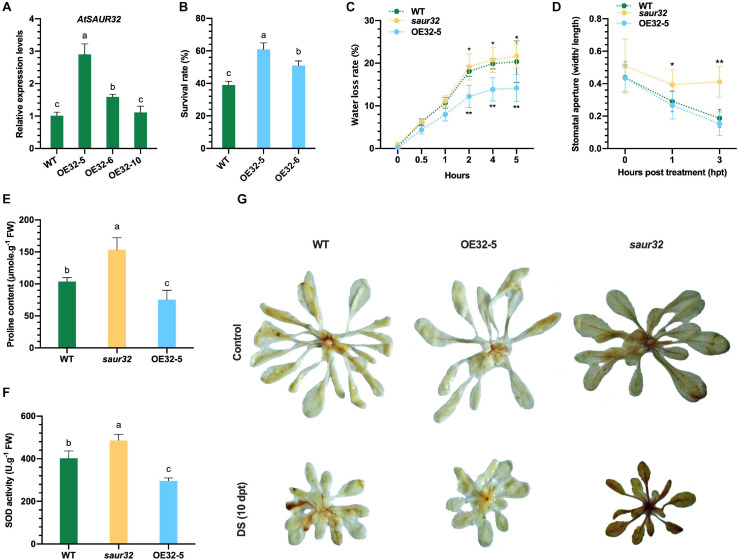
*AtSAUR32* loss-of-function and gain-of-function effect on Arabidopsis plant under drought stress conditions. **(A)** Transcript levels of *AtSAUR32* in wild type (WT) and *AtSAUR32*-overexpressed lines (OE32-5, OE32-6, and OE32-10). **(B)** Survival rates of WT and *AtSAUR32*-overexpressed lines (OE32-5 and OE32-6), 1 week after rewatering. **(C)** Transpiration water loss from WT, *saur32*, and OE32-5 Arabidopsis plant leaves at various time points after detachment. Leaves of similar developmental stage were detached and weighted at the mentioned time; leaves’ initial weight was used as a fresh weight to calculate water loss. **(D)** Stoma aperture of leaves of WT, *saur32*, and OE32-5 Arabidopsis plants under 10 μM ABA treatment. **(E)** Proline accumulation. **(F)** SOD content in WT, *saur32*, and OE32-5 plants. **(G)** Phenotypes showed DAB (3,3-diaminobenzidine) staining, indicating H_2_O_2_ accumulation after drought treatment. Mean values and standard deviation (+SD) were plotted as analyzed by Duncan’s multiple range (DMR) test (*P* < 0.05); lowercase letters (a–d) and asterisk (*) indicate significant difference.

To confirm the positive role of the drought stress-responsive *AtSAUR32* gene, it was overexpressed in Arabidopsis under the control of cauliflower mosaic virus 35S promoter. The 35S:*AtSAUR32* overexpressed lines were confirmed by PCR. To choose the best drought-resistant homozygous T_4_ lines for further investigation, we detected the relative expression level of *AtSAUR32* in WT and three selected *AtSAUR32*-overexpressed (*AtSAUR32*-OE) Arabidopsis lines (OE32-5, OE32-6, and OE32-10) under normal growing conditions. As presented in Figure 3A, the transcript level of *AtSAUR32* in OE32-5 and OE32-6 lines was substantially higher compared with WT and OE32-10. Thus, the OE32-5 and OE32-6 were selected for further investigation.

To determine drought tolerance, *AtSAUR32*-overexpressed (OE32-5 and OE32-6) and WT plants were grown for 4 weeks under normal conditions, then subjected to drought stress for 15 days, and rewatered, and survival percentage was estimated 7 days after rewatering. Results revealed that *AtSAUR32* overexpressed lines (OE32-5 and OE32-6) had significant higher survival rates than the WT plants (Figure 3B). Seven days after rewatering, the survival percentage in the OE32-5 line was > 60%, while the OE32-6 line had 50% and were screened out for further study. The transpiration rate was measured in detached rosette leaves to determine the degree of water retention following drought stress and found that the weight loss in the OE32-5 was consistently (2–5 h) significantly lower than in the leaves of *saur32* mutant and WT plants (Figure 3C), indicating that the increased drought resistance of OE32-5 plants might be due to reduced rates of leaf transpiration. Moreover, ABA signaling regulates drought response by regulating stomatal aperture. The stomatal aperture of the *saur32*, OE32-5, and WT plants leaves was measured after ABA treatment (10 μM). At 1 h ABA treatment, the stomatal aperture of *saur32* leaves was significantly enhanced (0.39) relative to WT (0.29), while OE32-5 leaves showed a slight reduction (0.26) (Figure 3D). However, with increasing treatment period, the stomatal aperture was significantly increased in *saur32* as compared with WT and OE32-5, which were 0.41, 0.15, and 0.18, respectively. These results are consistent with water loss assay (Figure 3C).

To evaluate physiological changes, the contents of free proline and SOD were measured following drought stress. In the *AtSAUR32* transgenic line OE32-5, free proline concentrations were approximately one half lower than those in the *saur32* mutants (Figure 3E), while in WT plants, the accumulation was 32% lower. Comparison of SOD contents in *AtSAUR32* transgenic and *saur32* mutant plants showed remarkably lower (38.9%) levels in OE32-5 transgenic plants relative to *saur32* mutant, while WT is 17.2% lower (Figure 3F). The higher accumulation of proline and SOD in the *saur32* mutants revealed that the effect of drought stress on *saur32* mutants was significant as compared with the WT and OE32-5 line, which retained their ability to curb the drought stress. Moreover, as an important indicator of hydrogen peroxide (H_2_O_2_) production/damage, DAB staining was carried out. Clearly, more intense DAB staining (brownish color) was observed in *saur32* mutant plant after 10 days of drought stress, which indicated H_2_O_2_ accumulation (Figure 3G). On the contrary, OE32-5 and WT plants showed lower accumulation of H_2_O_2_, which displayed minimum damage under stress conditions. Surprisingly *AtSAUR32* plays a negative role in the development of plant considering that the size, number, and fresh weight of leaves were significantly higher in the *saur32* mutant plants than those in the WT plants throughout the period of investigation, although comparable values were observed for fresh leaf weight at 30 days after germination (Supplementary Figure 4).

### AtSAUR32 Increases Sensitivity to ABA Responses

**FIGURE 4:**
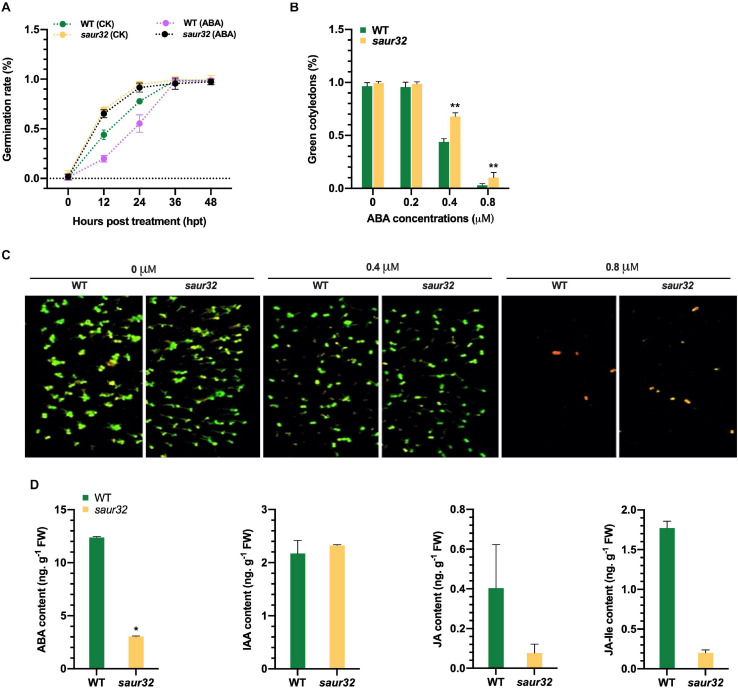
Effect of exogenous ABA application on WT, *saur32*, and OE32-5 plants. **(A)** Effect of ABA treatment (1 μM) on the germination rate of *saur32* mutant and WT; 0 μM ABA was used as control. **(B)** Effect of ABA concentrations (0, 0.2, 0.4, and 0.8 μM) on green cotyledons of WT and *saur32* at 5 days post-germination. **(C)** The cotyledons’ growth was observed in the 1/2 MS medium with 0, 0.4, and 0.8 μM ABA using Pulse-Amplitude-Modulation (PAM) chlorophyll fluorometer (Note: 0.2 μM ABA phenotypes are not added because phenotypically they are the same as 0 μM and have no significant difference). **(D)** Accumulation of endogenous hormones in *saur32* and WT plants. Mean values and standard deviation (+SD) are plotted as analyzed by Duncan’s multiple range (DMR) test (*P* < 0.05); asterisk (*) indicates significant difference.

The phytohormone ABA plays a prominent role in the development of plant and generally regulates drought response (Zhu et al., 2020). We detected the seed germination rate of *saur32* mutant and WT in the presence of ABA (1 μM) and mock media (0 μM ABA). The germination rate of *saur32* was substantially higher than WT on the medium containing ABA at 12 and 24 hpt, which indicated that *saur32* plants are insensitive to exogenous ABA application (Figure 4A). However, in later hours (36 and 48 h), the effect was decreased and no obvious differences were noticed in treated and untreated seeds. Further, different concentrations of ABA (0, 0.2, 0.4, and 0.8 μM) were used to assess the green cotyledon of *saur32* and WT plants. After 10 days of treatment, the WT seedlings showed a dramatic decrease in green cotyledon percentage in media supplemented with 0.4 and 0.8 μM ABA as compared with *saur32* seedlings (Figures 4B,C). However, PAM chlorophyll fluorometer results displayed a remarkable phenotypic difference between WT and *saur32* seedlings grown on medium with 0.8 μM ABA while those on 0 and 0.2 μM had no significant difference phenotypically (Figure 4C). These results indicated that *AtSAUR32* had a negative function in green cotyledon and seedling growth under ABA treatment. Together, these results implied that *AtSAUR32* significantly alters some physiological processes, confirming its active roles in Arabidopsis growth and development. Additionally, investigation of the physiological basis revealed that accumulation of ABA was significantly lower (fourfold) in *saur32* mutants compared with WT while the indole acetic acid (IAA) level was barely changed in both lines (Figure 4D). Although there were reductions in JA and JA isoleucine (JA-Ile) contents in saur32, no substantial differences were observed.

### RNA-Seq and Expression Analysis of Stress-Related Genes

In order to investigate defense-responsive genes from the transcriptomes of *saur32* mutant and WT plants, total RNAs were isolated and sequenced using an RNA-seq approach. A total of 124 DEGs were identified in which 102 were up-regulated while 22 were down-regulated (Supplementary Table 8). These genes were subjected to KEGG pathway analysis and the mainly enriched pathways associated with these DEGs included “plant hormone signal transduction” (ko04075), “nitrogen metabolism” (ko00910), “alanine, aspartate and glutamate metabolism” (ko00250), and so on. Besides, the transcription of some stress-responsive and phytohormone-related genes including *NAC29*, *bHLH100*, *WRKY40*, *SAG13*, *HSP70*, *HSP20*, *HSP20*, *JAZ23*, *ARR6*, *ARR15*, *HAI1*, and *AIP1* was alerted. We confirmed the expression of these DEGs through qRT-PCR analysis and similar patterns of expression were observed as exhibited in RNA-seq (Supplementary Figure 5). Furthermore, the functions of the DEGs were classified according to GO classifications. In GO annotations, most of them were enriched in cell, cell parts, binding, catabolic activity, cellular process, and single-organism process (Supplementary Figure 6).

**FIGURE 5:**
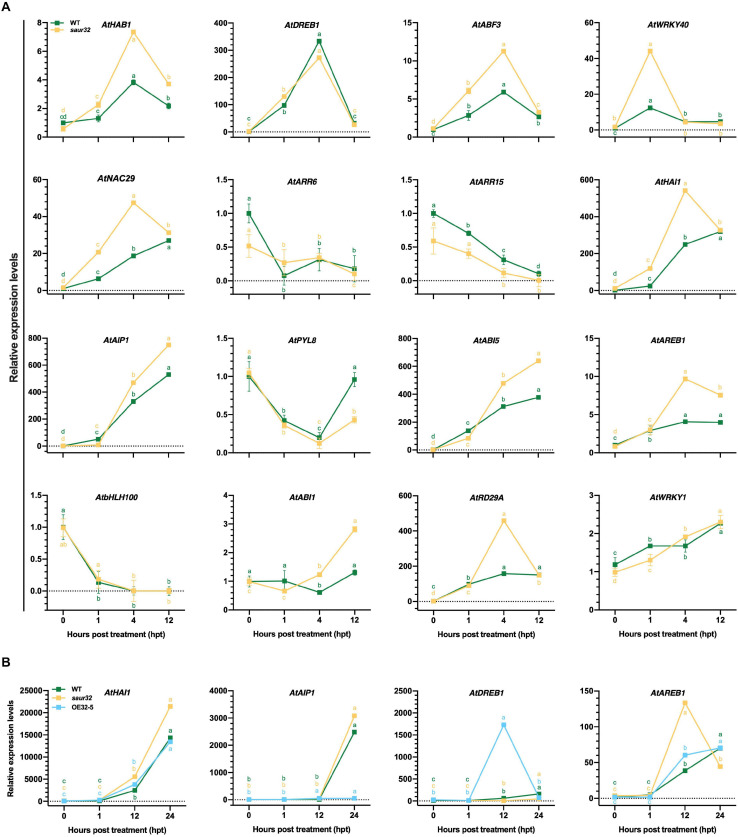
The expression levels of different genes in response to ABA and drought stress treatment. **(A)** Transcript levels of different genes during ABA treatment in WT, *saur32* plants. **(B)** Expression of genes under drought stress in WT, *saur32*, and OE32-5 plants. Mean values and standard deviation (+SD) plotted as analyzed by Duncan’s multiple range (DMR) test (*P* < 0.05); lowercase letters (a–d) indicate significant difference.

Further, *saur32* and WT plants were treated with ABA to examine the transcript levels of defense and ABA-responsive genes through qRT-PCR analysis. The analysis demonstrated that distinctive responses were observed in different genes (Figure 5A). Among all 16 genes, 9 were up-regulated in most of the tested time points in *saur32* relative to WT plants. Additionally, *DREB1* exhibited a remarkable increase in transcript level in WT (333) at 4 hpt than *saur32* (272). The *HAI1* and *AIP1*, which are clade-A PP2C genes and actively participate in ABA signal transduction, were significantly expressed in *saur32* plants as compared with WT during ABA treatment. So, the PP2Cs were found to be greatly induced in *saur32* relative to WT. Moreover, four genes were selected on the basis of their strong response to ABA and subjected to drought stress to quantify their transcripts by qRT-PCR. The analysis displayed that the expression of *HAI1*, *AIP1*, and *AREB1* was substantially induced by drought treatment in all lines (WT, *saur32*, and OE32-5), while in *saur32* mutants, the expression was high at several time points (Figure 5B). As a drought marker transcription factor, *DREB1* was greatly induced by drought stress in OE32-5 compared with WT and *saur32* at 12 hpt. Thus, the variation in expression levels of key genes was closely linked with drought tolerance of the *saur32* mutant.

### AtSAUR32 Protein Interacts With PP2C.A

**FIGURE 6:**
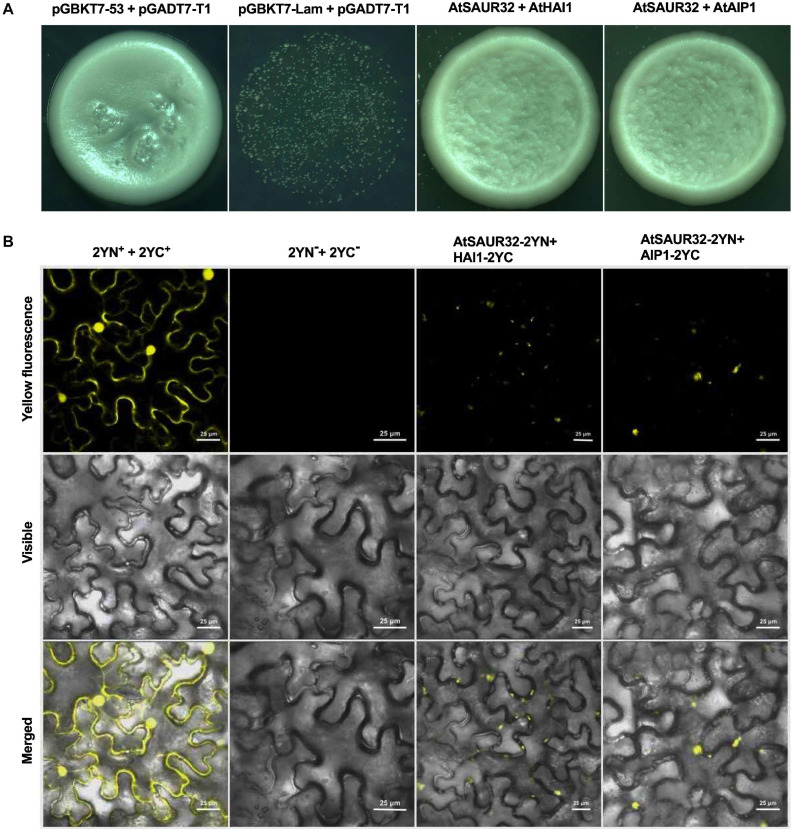
Identification of AtSAUR32 interacting proteins in Arabidopsis and their colocalization analysis. **(A)** Interactions of AtSAUR32 proteins with two PP2C.A (HAI1 and AIP1) in yeast two-hybrid assay. Growth of transformants on SD/-Leu-Trp-His-Ade indicates interaction. **(B)** For bimolecular fluorescence complementation (BiFC) analysis, plasmids were co-infiltrated into *Nicotiana benthamiana* and were observed after 48 h of agroinfiltration under a confocal microscope.

RNA-seq and expression data demonstrated that the transcript of ABA-responsive genes *HAI1* and *AIP1*, members of PP2C.A family, was significantly induced in the *saur32* mutant. So, we carried out Y2H assay to verify the potential AtSAUR32-interacting partners. The results showed that the yeast strain containing AtSAUR32-HAI1 and AtSAUR32-AIP1 proteins grew well in the selection media SD/-Leu-Trp-His-Ade, indicating strong protein–protein interaction (Figure 6A). To further confirm whether AtSAUR32 and PP2C.A genes (*HAI1* and *AIP1*) physically interact, we performed BiFC assay. AtSAUR32 infused with the N-terminal fragment of yellow fluorescence protein (YFP) could be co-expressed with AIP1 and HAI1, which were infused with the C-terminal, to produce the AtSAUR32-2YN+HAI1-2YC and AtSAUR32-2YN+AIP1-2YC vector. These vectors were cotransformed into tobacco cells, resulting in strong fluorescence signals. Confocal laser scanning microscopy displayed fluorescence at the nucleus, indicating localization to the nucleus of the cell (Figure 6B) and confirming that the AtSAUR32-HAI1 and AIP1 interaction occurred *in vivo*. Both Y2H and BiFC results indicated that AtSAUR32 actually could interact with AIP1 and HAI1 proteins.

## Discussion

Plants recruit various protective strategies to adjust themselves to unfavorable environmental conditions to complete the life cycle. Additionally, plants sense internal and external stress signals and induce a defensive system to sustain growth and adaptation. Among the different proteins involved in the defense system, SAUR proteins greatly share a common function in cell elongation, induce plant growth by regulating cell wall acidification, and regulate growth dynamically in response to environmental cues (Stortenbeker and Bemer, 2019). For example, Arabidopsis transcriptional profiles showed that the SAUR genes are strikingly induced by the growth-inhibiting hormone ABA (Ren and Gray, 2015; Qiu et al., 2020). However, the present study revealed that the SAUR family member *AtSAUR32* plays an important role in drought tolerance via ABA signal transduction, and it seems promising to identify a new player in ABA pathways.

The protein sequence of SAUR32 is highly conserved within the A1 subgroup of the SAUR family due to the presence of auxin_inducible domain and sequence similarities (Wu et al., 2012). As shown in Figure 1A, the phytohormones including ABA responsiveness elements (ABRE) were determined in the promoter of *AtSAUR32*, and several abiotic stressor responsive elements were also found such as MYB binding site (MBS), which is drought-inducible, LTR (low-temperature responsiveness), and TC-rich repeats (defense and stress responsiveness). The transcriptomic analysis displayed a significant increase in the transcript of *AtSAUR32* to drought and ABA, which were further confirmed by qRT-PCR analysis (Figure 1), while in contrast, some other SAUR genes were repressed by the application of ABA (Kodaira et al., 2011; Ren and Gray, 2015). Altogether, these outcomes demonstrate that our target gene has the potential to participate in different mechanisms in plants.

Combining studies of genome, proteome, and metabolome carried out over the last decade have revealed that SAURs regulate plant developmental and physiological processes, including leaf growth and senescence, hypocotyl growth, floral organ expansion, root growth, and development (Chae et al., 2012; Li et al., 2015; Ren and Gray, 2015). The tissue-specific expression results showed that *AtSAUR32* was highly expressed in different organs of the plant under normal growing conditions, suggesting that it plays a vital role in plant development (Figure 1B). Another study discovered that *SAUR36* is regulated by both auxins and gibberellins while its overexpression increased hypocotyl growth in light-growth conditions (Stamm and Kumar, 2013). These findings suggest that Arabidopsis SAUR proteins in different subfamilies may function in a similar manner and influence plant growth in various aspects.

The plant hormone ABA has dual functions as a growth inhibitor or stress indicator and is considered as a major signaling molecule in abiotic stress response (Gomez-Cadenas et al., 2015; Wang et al., 2018). ABA simultaneously controls stomatal closure and lateral bud dormancy, thus confirming the sensitivity to ABA of these traits (Shimizu-Sato and Mori, 2001; Arend et al., 2009). In the current study, loss of function of *AtSAUR32* weakened ABA sensitivity by increasing stomatal aperture and green cotyledon percentage as compared with WT and the *AtSAUR32* transgenic line (Figures 3D, 4B). Additionally, the seed germination of the *saur32* mutant was accelerated compared to that of control, though the seeds of *saur32* were insensitive to ABA during germination (Figure 4A). These findings implied that *AtSAUR32* probably participates in seed germination and stomatal movement by interaction with ABA signal transduction.

*AtSAUR32* overexpression enhanced drought tolerance in Arabidopsis while *AtSAUR32* knockout plants displayed contrasting results. Under drought conditions, OE32-5 had a higher *AtSAUR32* expression level, survival rate, and lower water loss rate, compared with WT and the *saur32* mutant (Figures 3A–C). During water-deficit conditions, cell membrane injury occurred and subsequently membrane lipid peroxidation was remarkably enhanced (Shi et al., 2014). We observed that the degree of cell membrane damage for *AtSAUR32* knockout plants was higher and their photosynthetic efficiency rate was on decline with increase in time (Figures 2E,F). Drought stress can also trigger the accumulation of reactive oxygen species (ROS), and SOD is the first defense line of action of the plant ROS defense (Huang et al., 2016). *AtSAUR32* knockout (*saur32* mutant) exhibited a higher level of osmotic regulatory substance (proline) content and SOD activity to quickly overcome stress and increase cell membrane stability (Figures 3E,F), thereby proving defensive reaction under stress (Ighodaro and Akinloye, 2018; Mohseni et al., 2018). As demonstrated in our study, loss of function of *AtSAUR32* decreased defense response to drought stress, which may be associated with higher SOD activity in the initial stage of ROS scavenging; similar findings were also noticed by Wang et al. (2016). Later, the over-accumulation of H_2_O_2_ occurred in the *saur32* mutant relative to WT and OE32-5 plants under drought conditions (Figure 3G), which exhibited that the *saur32* mutant suffered more seriously under drought while *AtSAUR32* (OE32-5) plays a positive role in stress. Therefore, the content of H_2_O_2_ is often used as an indicator of the level of damage to plant cells (Li et al., 2018). Proline is one of the most essential compatible osmolytes for cellular osmotic adjustment in many plant species during drought stress, high salinity, and other environmental stressors (Szabados and Savouré, 2010; Kumar et al., 2017). Additionally, the accumulation of proline eliminates the damage caused by ROS (Schöffl et al., 1999). So, the overall results showed that *AtSAUR32* could enhance drought tolerance to a significant level.

*AtSAUR32* probably regulates drought tolerance mediated by ABA-independent pathways and other drought-responsive factors. RNA-seq and qRT-PCR analysis demonstrated that the expression of some stress-responsive genes such as *AtNAC29*, *AtWRKY40*, *AtbHLH100*, *AtARR15*, *AtSAG13*, *AtJAZ23*, *AtOSM34*, *AtHSP20*, and *AtHSP70* showed obvious changes in the *saur32* mutant (Figure 5A and Supplementary Figure 5). *AtNAC29* and *AtWRKY40* were notably induced by ABA, while *AtbHLH100*, *AtARR6*, and *AtARR15* were severely repressed by ABA in *saur32* compare to WT. These genes probably participated in ABA and drought stress responses. Arabidopsis *AtWRKY40* was proved to be a negative regulator of ABA responses and bound to the promoters of multiple stress-inducible transcription factor genes and repressed their expression at low ABA concentrations (Xu et al., 2006). NAC transcription factors play an important role in stress response via both ABA-independent and ABA-dependent pathways. Thus, *NAC016*-, *NAC019*-, *NAC055*-, and *NAC072*-overexpressed plants exhibit strong drought tolerance, indicating that NAC TFs positively regulate drought stress-responsive signals (Fujita et al., 2004; Tran et al., 2004). The *ANAC072*/*RD26* overexpression trigger ABA sensitivity, though transgenic plants in which its activity was repressed were insensitive (Nakashima et al., 2012). The transcript levels of *bHLH122* were strongly induced by drought, and up-regulation of *bHLH122* substantially increased cellular ABA levels, which demonstrated that *bHLH122* functions as a positive regulator of water-deficit conditions (Liu et al., 2014). Heat shock proteins (HSPs) are induced by drought and correspond to enhanced tolerance (Aneja et al., 2015). Previous studies found that overexpression of *HSP17.6A* in Arabidopsis resulted in gain of tolerance in these plants during water-deficit conditions, possibly through protein stabilization (Sun et al., 2001). Hence, the above results suggest that *AtSAUR32* positively participates in drought response via ABA-independent and ABA-dependent pathways, while these findings will help to understand the complex auxin and ABA signaling interaction network.

**FIGURE 7:**
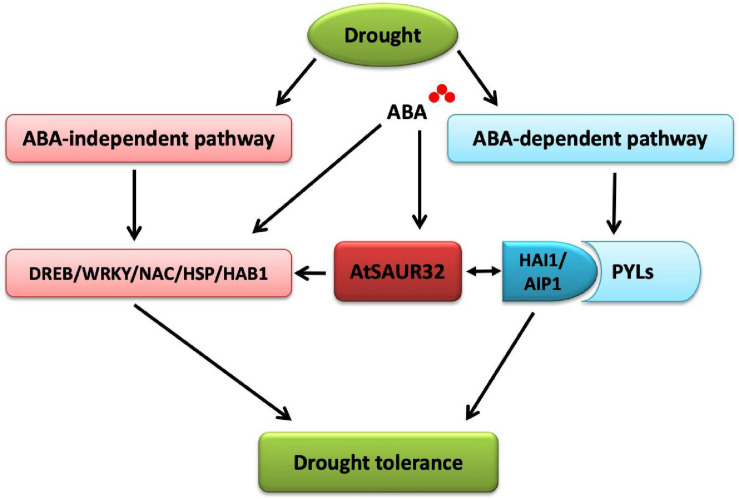
Proposed model for the roles of *AtSAUR32* in drought stress resistance in Arabidopsis.

Plant reduces water loss by ABA accumulation, which triggers ABA-dependent signaling that causes the activity of clade-A PP2Cs such as HAI1 and AIP1, to be inhibited through their interaction with ABA stimulated PYL. This, in turn, allows SnRK2 kinase to regain activity and phosphorylates its targets such as ABF transcription factors that control ABA-dependent gene expression and influence stomatal aperture. As clade-A PP2Cs, the HAI1 gene has a more prominent role in drought response compared to AIP1; however, HAI mutants enhanced proline and osmoregulatory solute accumulation at low water potential (Bhaskara et al., 2012). The interactive behavior of HAI1 with PYL5 and PYL7–10 of the PYL/RCAR ABA receptor family participates in feedback regulation of ABA signaling, which is quite possible due to dephosphorylation of SnRK2s (Fujita et al., 2009; Antoni et al., 2012). In our study, the PP2C.A subfamily genes including *HAI1* and *AIP1* were identified as DEGs using RNA-seq analysis and qRT-PCR analysis. Furthermore, the expression of *HAI1* and *AIP1* was up-regulated and was notably induced by ABA and drought stress in the *saur32* mutant (Figures 5A,B). The expression pattern of some ABA and drought marker genes such as *AREB1*, *ABF3*, and *DREB1* was altered under ABA and drought treatment. Meanwhile, Y2H and BiFC analyses indicated that HAI1 and AIP1 proteins highly interacted with AtSAUR32 (Figures 6A,B); however, further work may help to support the potential *in vivo* interaction of SAUR32 with these phosphatases. A previous study showed that AtSAUR19 subfamily proteins were upstream regulators and interacted to repress the expression of clade-D PP2Cs. Here, AtSAUR32 is speculated to interact with clade-A PP2Cs participating in the model of ABA-dependent PYL-PP2C-SnRK2s interaction in ABA signaling (Figure 7).

## Conclusion

In conclusion, *AtSAUR32* transcription is sensitive to exogenous ABA application and enhances drought resistance via ABA signal transduction. The physiological and morphological attributes implied that *AtSAUR32* is more sensitive to ABA; however, under drought stress conditions, the *saur32* mutant suffered more seriously as compared with WT and OE32-5 plants. So, *AtSAUR32* might be positively involved in response to drought conditions; however, further genetic analyses would help to confirm these results. Moreover, RNA-seq and qRT-PCR analysis identified the co-regulatory defensive genes, PP2C.A subfamily members that were remarkably induced by ABA and drought in *saur32* relative to WT. Y2H and BiFC analysis revealed that some PP2C.A protein AHI1 and AIP1 interacted with AtSAUR32. Considering that, PP2C.A genes in Arabidopsis might be regarded as key regulators of ABA signal transduction, while in drought response, AtSAUR32 was found to mediate ABA-dependent signal transduction through AHI1 and AIP1. Besides, the transcripts of stress-responsive genes (*WRKY40*, *NAC29*, *bHLH100*, and *HSPs*) were altered, and their response patterns to ABA were changed with loss of function of *AtSAUR32*. Put together, these results suggest that *AtSAUR32* probably regulates drought tolerance through ABA-dependent and -independent pathways.

## Data Availability Statement

The datasets presented in this study can be found in online repositories. The names of the repository/repositories and accession number(s) can be found in the article/ Supplementary Material.

## Author Contributions

GL and YH conceived the study. YH, YL, and ML generated transgenic lines and performed phenotypical observation. YH, DY, and AL-S carried out the cytological analysis. MA, YH, MI, IJ, and XY performed the data analysis. MA and GL wrote and revised the manuscript. All authors discussed and commented on the final manuscript.

## Conflict of Interest

The authors declare that the research was conducted in the absence of any commercial or financial relationships that could be construed as a potential conflict of interest.

## Footnotes

1 https://www.arabidopsis.org/index.jsp

2 http://bioinformatics.psb.ugent.be/webtools/plantcare/html/
