# Supplementary material for: The Arabidopsis SMALL AUXIN UP RNA32 Protein Regulates ABA-Mediated Responses to Drought Stress
Source: Front Plant Sci. 2021 Mar 12;12:625493. doi: 10.3389/fpls.2021.625493 (PMC7994887; doi:10.3389/fpls.2021.625493)
Supplement: Supplementary file 1 [file Data_Sheet_1.docx]

**The Arabidopsis SMALL AUXIN UP RNA32 protein regulates ABA-mediated responses to drought stress**

Yanjun He^1†^, Yue Liu^1†^, Mengzhuo Li^1^, Anthony Tumbeh Lamin-Samu^1^, Dandan Yang^1^, Xiaolin Yu^1^, Muhammad Izhar^2^, Ibadullah Jan^3^, Muhammad Ali*^1^, Gang Lu*^1,4^

^1^Department of Horticulture, College of Agriculture and Biotechnology,Zhejiang University, Hangzhou 310058, China

^2^College of Agronomy, Northwest A&F University, Yangling, China

^3^Department of Agriculture, University of Swabi, Khyber Pakhtunkhwa, Pakistan

^4^Key Laboratory of Horticultural Plant Growth, Development and Quality Improvement, Ministry of Agricultural, Zhejiang University, Hangzhou 310058, China

^†^These authors contributed equally to this work

*Corresponding Authors: [maur202@zju.edu.cn](mailto:maur202@zju.edu.cn) and [glu@zju.edu.cn](mailto:glu@zju.edu.cn)

**
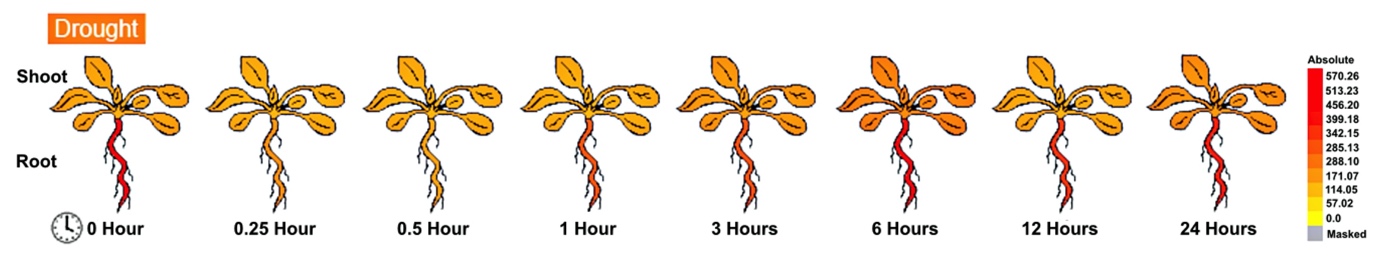
**

**Figure S1** Predicted transcript levels of *AtSAUR32* in shoot and root under drought condition. Scale bar ranged from yellow (0) to red (570); data were retrieved from TAIR website.

**
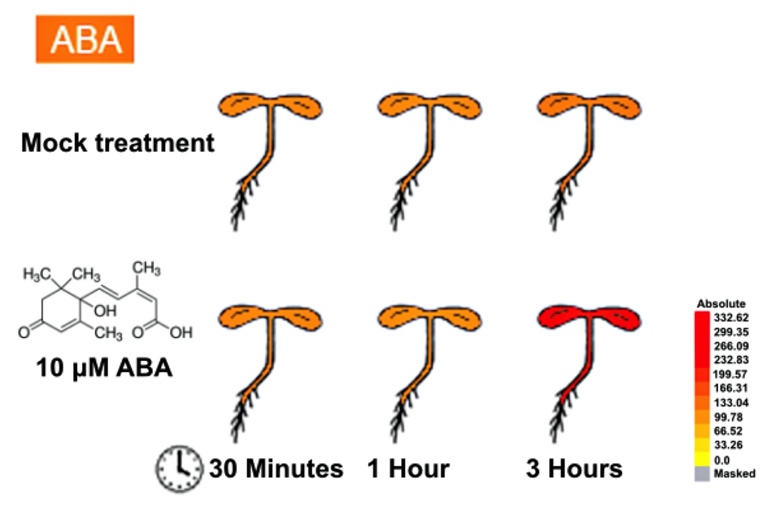
**

**Figure S2** Predicted expression levels of *AtSAUR32* in Arabidopsis shoot and root under 10 μM ABA treatment. Scale bar ranging from yellow (0) to red (332); data were retrieved from TAIR website.

**
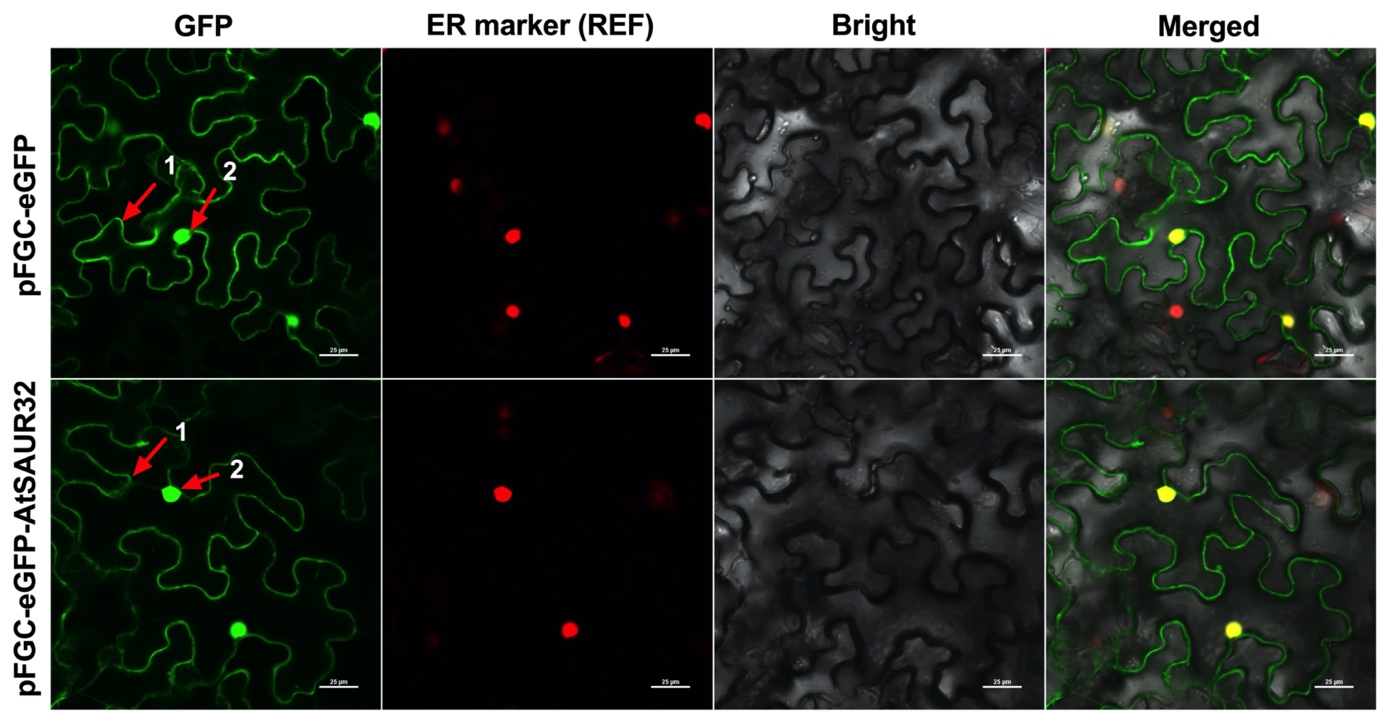
**

**Figure S3** Protein localization assay of AtSAUR32 in tobacco leaves epidermal cells. 35S::GFP (green ﬂuorescence protein) represents control. A bright ﬂuorescent ﬁeld was used to measure the ﬂuorescence. The numbers 1 and 2 represent the nucleus and cell membrane, respectively.

**
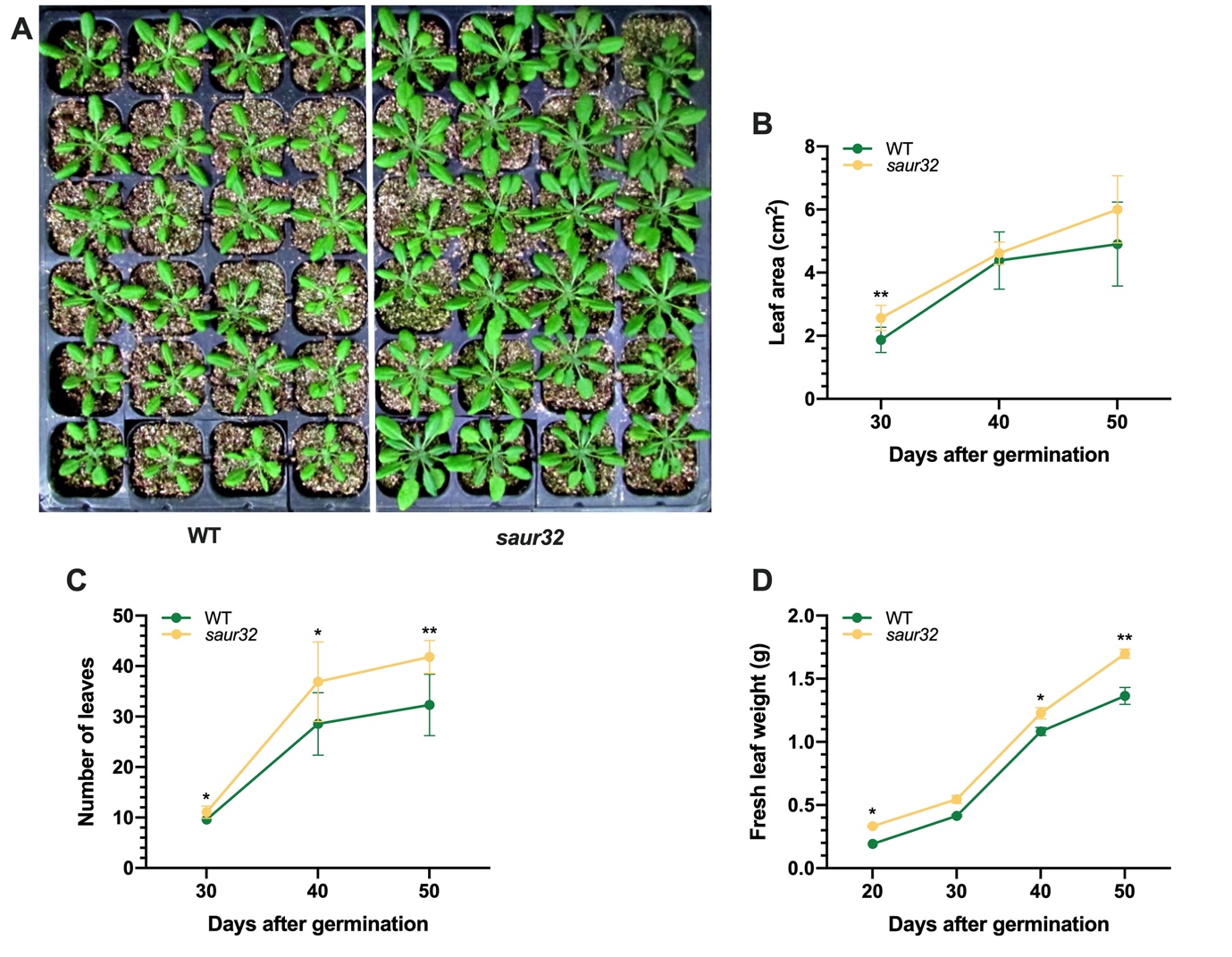
**

**Figure S4** Effect of *AtSAUR32* knockout on the development of Arabidopsis plants **(A)** The phenotypes of wild-type (WT) and *saur32* mutant under normal condition **(B−D)** Leaf size, number of leaves and fresh leaf weight of WT and *saur32* mutant. Mean values and standard deviation (+SD) plotted as analyzed by Duncan’s multiple range (DMR) test (P<0.05); asterisk (*) indicate significant difference.

**
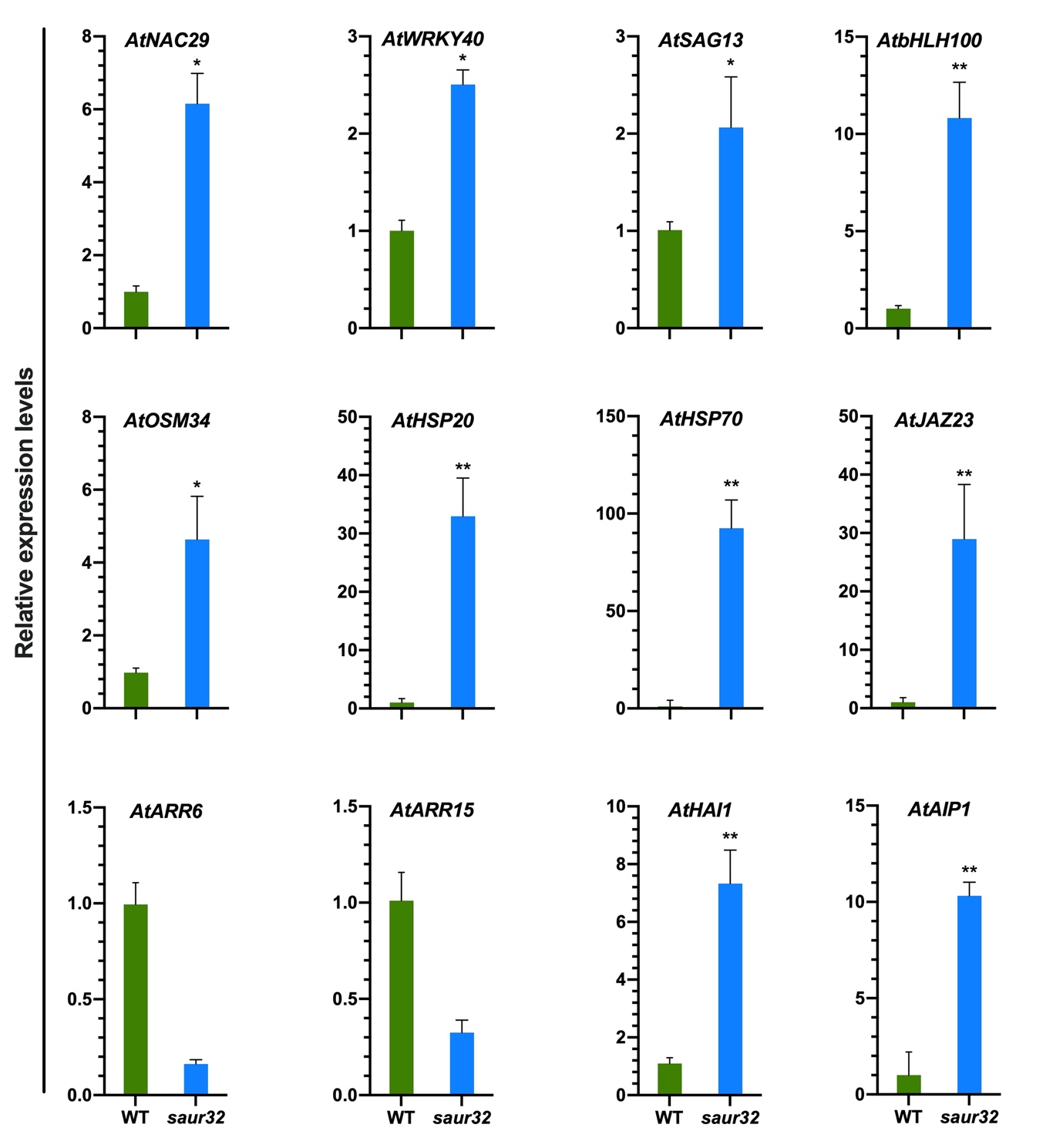
**

**Figure S5** The qRT-PCR analysis of different genes in wild-type (WT) and *saur32* mutant plants under normal conditions. Mean values and standard deviation (+SD) plotted as analyzed by Duncan’s multiple range (DMR) test (P<0.05); asterisk (*) indicate significant difference.

**
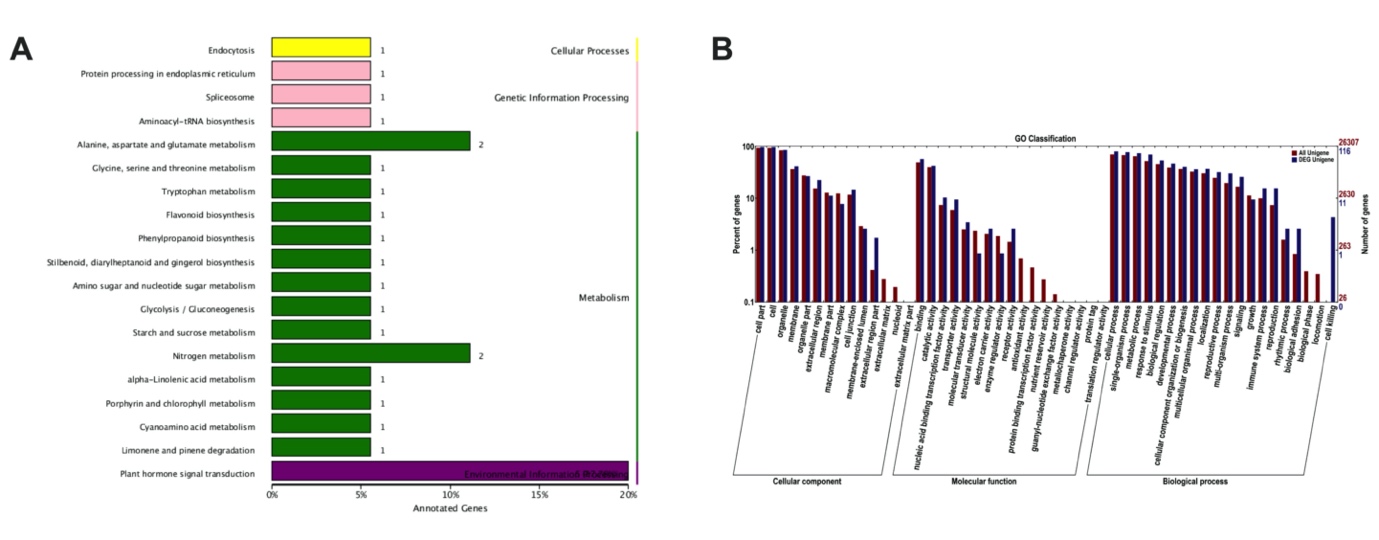
**

**Figure S6** RNA-seq analysis of *saur32* mutant plants **(A)** ﻿Functional analysis of differentially expressed genes (DEGs) based on RNA-Seq data in different comparisons **(B)** Gene ontology analysis in three categories (cellular component, molecular function and biological process).
