# Supplementary material for: The Arabidopsis SMALL AUXIN UP RNA32 Protein Regulates ABA-Mediated Responses to Drought Stress
Source: Front Plant Sci. 2021 Mar 12;12:625493. doi: 10.3389/fpls.2021.625493 (PMC7994887; doi:10.3389/fpls.2021.625493)
Supplement: Supplementary file 2 [file Data_Sheet_2.docx]

**The Arabidopsis SMALL AUXIN UP RNA32 protein regulates ABA-mediated responses to drought stress**

Yanjun He^1†^, Yue Liu^1†^, Mengzhuo Li^1^, Anthony Tumbeh Lamin-Samu^1^, Dandan Yang^1^, Xiaolin Yu^1^, Muhammad Izhar^2^, Ibadullah Jan^3^, Muhammad Ali*^1^, Gang Lu*^1,4^

^1^Department of Horticulture, College of Agriculture and Biotechnology, Zhejiang University, Hangzhou 310058, China

^2^College of Agronomy, Northwest A&F University, Yangling, China

^3^Department of Agriculture, University of Swabi, Khyber Pakhtunkhwa, Pakistan

^4^Key Laboratory of Horticultural Plant Growth, Development and Quality Improvement, Ministry of Agricultural, Zhejiang University, Hangzhou 310058, China

^†^These authors contributed equally to this work

*Corresponding Authors: [maur202@zju.edu.cn](mailto:maur202@zju.edu.cn) and [glu@zju.edu.cn](mailto:glu@zju.edu.cn)

**Table S1 *AtSAUR32* (AT2G46690) promotor, DNA, CDS and protein sequence**

| **Promotor: Upstream 3000bp** | |
| --- | --- |
| CTATGAGTTTATAAGAATTTGGTTAGACAGACTTATTTAAATGTTTTATTTTCGGATTTTCCTTACAATTTATTTTTTTCCCATATAATTTATTTGTCGAAATTCCCATATATATTATACACATTTTTAAGAAATAAAATATATTGCCGACAAAAAAAAAAAAAAAAAATTATTGATGACCATGGGCTTTAATTGGGCTTATACAAGTCATCAATTTGACAGCCGATAACGGTTAATTAACAGAAGCCCATGATAGTTTCCGTGGTTTTTATATCTACGAACTTATCACTAATATATTATACACACATTTGGCCCATTGGTATTTGACAGTGTCTCGTGAATTTGTTTTTCAAAGAATCAAAAGATAAATAATTGGTGACAAATAGCATGCATTATTTGCTATATAAAAAAACTATAGCATGAATTATAGTTTTGACTGTAATACTGTATATTGACCAAAAATCAAATTATCTGTAAAATATGTGAAATGAAATCATGAATAAAAACGGAAACTTATTTGTAAATTCGCAAATGGGCATCATATATTTGTTGTCATGTTATAATTAATTTGCCAATTGACATGAGTCAAGACTGTACGATTATACGGTTCCGGTGTGGATGGTTCATCACACATGAGGGCACCCTATTAGAACCGGCTTTAGAATATGAAGATTTTGATGTTAAAAAGAAGGCTGTACCAATATACGATGATTTCTACAAACTCATATCAGGAAAATTATATTATCCTGCTGTTTTTTGCATGCGCAATCGAATAGTAGTTTTAAGTAAGAAAAATAGGGGAAATATAAATAGTAGTATAATCCAAGGTACCCACACCGTCCCACAAAGTTGACAATTTTTGAATATCGTTGTCTCATTTTTCACACAAAGTGATTATTGTTAGTGTGCACTGCTTTATCTTATCCTCTATAGACGATCAACTTTCATTTTCCTCTCTTATTTTATTAAAATTGAAAACGGATCTAACAAATTTTCATTTTTATTTCATTTATCTTCTGGTTCTAGATACTGATAGAGTGACATGTGCCTTTTCTTTTTACCTTTTAAAATTCATATATTTTTCTTTTCTCTGTACATAATAAGTATTCGCTACCATGTCACTACCACCAACTTGGACGGCCTCGTTTTTTATTCAACTCGTCAGGAAATTGATAACCATTTACTTGTTAAAGATATGATTATTGTATTCGTACGAATTCATAGATGTGGTACCATTGAGCATATCAATATTAGGCTAGAATATTCCTTCTCCAAATTTTGTGTGTTTGATGGCACAAATTTTAGTTGTTTTCATTAGTAACTGTCTTTACTTATTGGATTAGATAACATGGATTATAGGCTCCTCGAAGGTGCCAATATCCATCAACGCTTACATCGAAATTAAACCGGCCGGTTTATGCTAAAATCCGTTGACCCAATCGCCGTTATGCTAATGTTAAGTGACAATTCTAAATACGATAATGGAATGATTTTTAAATATAAACGTAGTTTATACCTAAAACAACAAAAAGAAAAAGTAGTTTATACGTGGACATGTCTTAGACCGGCTGCGTCTTGTTCATCGAATTGTCTCCGAATTTGGATCATTACATATCTATAACTGTATTTATAATAAACTTGTAAGTTTTAGATATCGACTTGCATAATGTGTATACATGAGCTCATAGATATTGCATATATACAAGCGATGTTTAAATTATTAAACAATCGCTATATATATACATCACCACCTCCAGTCTTCATCATGTGACGACATTGATTCTGTTTCTTTTATAACTAACAATACCTAAGCCGCTCCAATAATATATAAATAATTGGTAACTACAGTTAACAAAACGAAAGTCGCAGAGAGATCAACATTGTGACAATATTTAATAATGTAATTTCTCTTTGCCATTTAAGATTCCATATGACGACGGCTCCTTTATTTGGATATAAGTTTCAATTTACGCATATTTCCGACTATCAAACCTTTTCTCTTTGTAAGATGAATAGTTACACTGAATCCACCCAAATTTTATAGCTTTTCTTTTTGAATATATGAGAATAGTCAGTTTTGAATTTCCTTTGCATAGTTTGATCACGCTTTTAACGTTCTTAATTGATGATATATTTTTCGCTAGTAATACTCTTAGGTTTTAGGTAAAACAGTAATATGTCGACTAATTTGACTAATTTACTCGGTTTAGCACTTAGTTAAAATTTCAACCCTTTTTCTTGAGTCACCAACCCATTATTAACTGTTGGTCGTCATTACTCTTTAATTCTCAGTTGCTGACCACATACTTTCTTAAAACTATTTGTTGACGAGTAATTAAAACAATTTATGAGTATCTTTTACTTCATAAAAAATATTAAAAGGACAACTATATTAGGAAATATAAAAGAATATTAGTTATAAACTTACTGTCATATTGATATATAGCTATGCAAACAATAGAAAATAGATTCAGCATTCATTGGGTTTCTTAATTTAAGTGTTCTATATTTTTGGTAGTTCTCTTACCAATAGAATAACGTTAACAATATTTTTAATAAAAACATATACAGATATTTCTAAAGTCTTACACTTCATTATTTAGCATAATGTTTTGTTTTATATATTTGTAATATAAAAAAGTGCTACTCGATAGAGTTTCTCTTTAAATATTTTAGTTGATAATCCTTGAATTTTTCTTACTTATTGCTAATATTTTTGGTCAAATCTTAATTATTGCTAGTTACAAACTTCTTAAAATACCCATTGGTTATTATTAGATTTTATAAAGAAAATGACAACCAAATAGGAATATTTTTAATACCACCATTGTATTTATTAATGAGAAGTTGAATAAAAGAAAATAGGAGCGTCTCATCTCTTTTTGAAGGGCTTTTTTAAAAAACAAATACTAGTGTGTTTTCTCTTTATAAAACAAACCTCACTCTTTTGTTTCTTTCATTCATCATCTCCACACAAAAAAAAAAAAAAAAA | |
| **DNA sequence** | |
| AAAAAAAAAAAAAAAAAATGAACAACTTAATTAGCTCTCTCGAACTCACATCTCTTACACTTGAAGACCCATGGCCTGCTTTCTAGCCTAAATTTCCTCGAGAAAACAAGCAAAAAAACACAAGAAAGCTAGAATCTTATTTTCTTCTTGGTCTTTCTTGGTTTCTTCAACTTTTTCTTAAAAACGTTTTCAAAAAATCATGGGCACCGGAGAAAAAACCCTGAAGAGCTTCCAGTTACATCGCAAACAATCAGTCAAAGTCAAAGATGTTCCAAAAGGGTGTTTAGCGATCAAAGTGGGATCGCAAGGAGAAGAGCAACAGAGATTTATCGTTCCTGTTTTGTATTTTAACCATCCATTGTTCATGCAGCTCCTGAAAGAAGCAGAAGACGAGTATGGATTCGATCAAAAGGGCACCATCACAATTCCTTGTCACGTGGAGGAGTTTCGTTACGTTCAAGCTTTGATAGATGGAGAGAGATCAGTTTACAATGGTAACAACCATCATCATAGACATGGTGGCCGTGACCAGTATCATCATCTTGTTGGATGCTTCAGAGCTTGATGAATAATGATGAAATGAAATGATGGGTGGTGATTGTATGTGTTCAAATTTTAATGTTTTTTCTGGTTTAATCTTGTGGAGTTGTGGAGCCAAAACTGGCCTAACCCACAAAAGAAGTGAATCTTCTAAATTCTATCGATTTGTAGTATCTTTTTCTGTAATTTTAATGGAATTTTAAGATTTAAGAGTGTTGTTGTTTCTGATTTTTTTTCTTTTTCTTTTATTGCTTTAGACCATTTTTGAAAGGTTGTAGATAAACATGACTACGATGATCACAAAGAAACGAAAAAGATTGACACATTTTTTGGAAATTTGCAACTGTTAGACACGT | |
| **CDS sequence** | **Protein sequence** |
| ATGGGCACCGGAGAAAAAACCCTGAAGAGCTTCCAGTTACATCGCAAACAATCAGTCAAAGTCAAAGATGTTCCAAAAGGGTGTTTAGCGATCAAAGTGGGATCGCAAGGAGAAGAGCAACAGAGATTTATCGTTCCTGTTTTGTATTTTAACCATCCATTGTTCATGCAGCTCCTGAAAGAAGCAGAAGACGAGTATGGATTCGATCAAAAGGGCACCATCACAATTCCTTGTCACGTGGAGGAGTTTCGTTACGTTCAAGCTTTGATAGATGGAGAGAGATCAGTTTACAATGGTAACAACCATCATCATAGACATGGTGGCCGTGACCAGTATCATCATCTTGTTGGATGCTTCAGAGCTTGA | MGTGEKTLKSFQLHRKQSVKVKDVPKGCLAIKVGSQGEEQQRFIVPVLYFNHPLFMQLLKEAEDEYGFDQKGTITIPCHVEEFRYVQALIDGERSVYNGNNHHHRHGGRDQYHHLVGCFRA |

**Table S2 *saur32* mutant detection primers**

| LP： TTTCGTTTCTTTGTGATCATCG |
| --- |
| RP： TTTTTGGTCAAATCTTAATTATTGC |
| LBb1.3： ATTTTGCCGATTTCGAAC |

**Table S3 *AtSAUR32* overexpression primers**

| **Vector** | **Primer sequence (5’-3’)** | |
| --- | --- | --- |
| SAUR32 | F: | CACCTTTCTAGCCTAAATTTCCTCG |
|  | R: | GATTCACTTCTTTTGTGGGTT |

**Primers used for transgenic plant confirmation**

| **Vector** | **Primer sequence (5’-3’)** | |
| --- | --- | --- |
| SAUR32 | F: | AGTTCACCTTGATGCCGTTC |
|  | R: | GATTCACTTCTTTTGTGGGTTT |

**Table S4 Yeast-two-hybrid (Y2H) and bimolecular fluorescence complementation (BiFC) primers.**

|  | **Genes** | **Primer sequence (5’-3’)** | |
| --- | --- | --- | --- |
| Y2H | *AtSAUR32* | F: | CGGAATTCCAAAAAATCATGGGCACCGGAG |
|  |  | R: | TCCCCCGGGGAACACATACAATCACCACCC |
|  | *AtHAI1* | F: | CGGAATTCGATGGAGTGTGTTTGTTG |
|  |  | R: | TCCCCCGGGGAGAGACAAATACAACTACG |
|  | *AtAIP1* | F: | CGGAATTCGTTATGGCGGATATTTG |
|  |  | R: | TCCCCCGGGCGGTGGAGTAAGAGTGATG |
| BiFC | *AtSAUR32* | F: | CCTTAATTAAATGGGCACCGGAGAAAAAAC |
|  |  | R: | AGGCGCGCCAAGCTCTGAAGCATCCAAC |
|  | *AtHAI1* | F: | CCTTAATTAAATGGCTGAGATTTGTTACG |
|  |  | R: | AGGCGCGCCACTTCGTGTCTCGTCGTAGATC |
|  | *AtAIP1* | F: | CCTTAATTAAATGGCGGATATTTGTTA |
|  |  | R: | AGGCGCGCCAAGCAACGTGTCTC |

**Table S5 Subcellular localization of the AtSAUR32 primers**

| **Vector** | **Primer sequence (5’-3’)** | |
| --- | --- | --- |
| GFP-SAUR32 | F: | GCTGTACAAGGGATCCATGGGCACCGGAGAAAAA |
|  | R: | TAATTAACTCTCTAGATCAAGCTCTGAAGCATCCAACAAG |

**Table S7 qRT-PCR primers**

| **Genes** | **Primer sequence (5’-3’)** | |
| --- | --- | --- |
| *AtActin7* | F: | CCATTCAGGCCGTTCTTTC |
|  | R: | CGTTCTGCGGTAGTGGTGA |
| *AtSAUR32* | F: | GGAGTTTCGTTACGTTCAAGC |
|  | R: | CACATACAATCACCACCCATC |
| *ARR6* | F: | GTTATGCTACCGAGGAAGATG |
|  | R: | GGCAAGAACATGAAGAGGATC |
| *ARR15* | F: | AACAATGTATGATAGAAGGAGCAG |
|  | R: | GAGTGTCGTCATCAAGGGAG |
| *AtHSP20* | F: | AAGGCAGATTTCAGGGAC |
|  | R: | CAAGTCGACACGCACG |
| *HSP70* | F: | CCTCTGTCCTTGGGTTTG |
|  | R: | TTGTCCTTTGTTCGTGCC |
| *OSM34* | F: | ACTGTTGTACGAACGGTCAG |
|  | R: | AGTCGGGTCATCTTGTGG |
| *JAZ23* | F: | TCGTTATTGAGGATGGAGG |
|  | R: | CAGGCGGAGACGTTGTTAG |
| *SAG13* | F: | AGCGACAACATAAGGACG |
|  | R: | CAAAGAAATGCCACAAGC |
| *WRKY40* | F: | CACTACCCTCGTTGTGAAAG |
|  | R: | ATGGCATTGGATGGTTG |
| *bHLH100* | F: | CCGTGGTGATGAAGAAGC |
|  | R: | CGTTTGATTGGTGGGAGG |
| *NAC29* | F: | CAGACAAAGCCATTCACAG |
|  | R: | CAGTACCCATTCATCTAACCTC |

**Table S7 Function of the cis-elements found in the promoter region of *AtSAUR32***

| **Site name** | **Sequence** | **Function** |
| --- | --- | --- |
| ABRE | ACGTG | cis-acting element involved in the abscisic acid responsiveness |
| TC-rich repeats | GTTTTCTTAC | cis-acting element involved in defense and stress responsiveness |
| MBS | CAACTG | MYB binding site involved in drought-inducibility |
| LTR | CCGAAA | cis-acting element involved in low-temperature responsiveness |
| CGTCA-motif | CGTCA | cis-acting regulatory element involved in the MeJA-responsiveness |
| TCA-element | CCATCTTTTT | cis-acting element involved in salicylic acid responsiveness |

**Table S8 Total 124 differentially expressed genes (DEGs)**

| **SN** | **#ID** | **FDR** | **log2FC** | **Regulated** | **nr_annotation** |
| --- | --- | --- | --- | --- | --- |
| 1 | AT1G04570 | 0.00154085 | 1.46236067 | up | probable folate-biopterin transporter |
| 2 | AT1G07130 | 0.00307149 | 1.78442741 | up | STN1-like protein |
| 3 | AT1G07430 | 0.00632989 | 1.04640975 | up | protein phosphatase 2C 3 |
| 4 | AT1G11080 | 8.98E-32 | 1.79113878 | up | serine carboxypeptidase-like 31 |
| 5 | AT1G13609 | 0.00263987 | 2.95715643 | up | defensin-like protein 287 |
| 6 | AT1G14190 | 1.94E-06 | 3.64683271 | up | glucose-methanol-choline oxidoreductase-like protein |
| 7 | AT1G15380 | 7.58E-06 | 2.6352311 | up | Lactoylglutathione lyase / glyoxalase I family protein |
| 8 | AT1G17020 | 0.00348233 | 1.37383183 | up | Fe(II)/ascorbate oxidase family protein SRG1 |
| 9 | AT1G17960 | 0.00104858 | 2.32493248 | up | threonyl-tRNA synthetase |
| 10 | AT1G18400 | 1.25E-17 | -1.3581439 | down | transcription factor BEE 1 |
| 11 | AT1G19530 | 0.00099953 | 1.97700458 | up | uncharacterized protein |
| 12 | AT1G22550 | 1.69E-08 | -1.3054658 | down | putative peptide/nitrate transporter |
| 13 | AT1G26900 | 3.64E-08 | -3.4558493 | down | pentatricopeptide repeat-containing protein |
| 14 | AT1G52040 | 7.18E-53 | 1.06941233 | up | myrosinase-binding protein 1 |
| 15 | AT1G53480 | 9.44E-18 | #NAME? | down | mto 1 responding down 1 protein |
| 16 | AT1G53490 | 1.42E-83 | -4.8164094 | down | RING finger-containing protein HEI10 |
| 17 | AT1G56600 | 1.50E-36 | 1.73101407 | up | galactinol synthase 2 |
| 18 | AT1G60190 | 1.51E-67 | 2.17996554 | up | U-box domain-containing protein 19 |
| 19 | AT1G63350 | 2.72E-05 | Inf | up | CC-NBS-LRR class disease resistance protein |
| 20 | AT1G65890 | 0.00019012 | 1.50353852 | up | acyl activating enzyme 12 |
| 21 | AT1G68620 | 0.00227405 | 1.24108096 | up | probable carboxylesterase 6 |
| 22 | AT1G69490 | 2.07E-06 | 1.94410576 | up | NAC transcription factor protein family |
| 23 | AT1G70260 | 1.12E-26 | 1.70416298 | up | nodulin MtN21-like transporter UMAMIT36 |
| 24 | AT1G72830 | 0.00065131 | 1.23434706 | up | nuclear transcription factor Y subunit A-3 |
| 25 | AT1G73480 | 3.92E-45 | 1.17871384 | up | alpha/beta-Hydrolases superfamily protein |
| 26 | AT1G74750 | 0.00185589 | Inf | up | pentatricopeptide repeat-containing protein |
| 27 | AT1G74890 | 0.00014604 | -1.2508063 | down | two-component response regulator ARR15 |
| 28 | AT1G80130 | 2.23E-11 | 1.1587864 | up | tetratricopeptide repeat domain-containing protein |
| 29 | AT1G80160 | 1.20E-09 | 4.90983256 | up | GLYOXYLASE I 7 |
| 30 | AT1G80820 | 0.00751925 | 1.56237392 | up | cinnamoyl-CoA reductase |
| 31 | AT1G80840 | 0.00336344 | 1.02845122 | up | putative WRKY transcription factor 40 |
| 32 | AT2G04050 | 1.51E-15 | 3.13456859 | up | MATE efflux family protein |
| 33 | AT2G04070 | 0.00325633 | 2.42938563 | up | MATE efflux family protein |
| 34 | AT2G05440 | 2.42E-34 | 1.42612273 | up | glycine-rich protein 9 |
| 35 | AT2G13600 | 3.38E-08 | -3.6257534 | down | protein SLOW GROWTH 2 |
| 36 | AT2G14247 | 1.96E-12 | 2.720898 | up | uncharacterized protein |
| 37 | AT2G16586 | 5.57E-13 | 1.34815938 | up | uncharacterized protein |
| 38 | AT2G18050 | 1.38E-22 | 1.70528852 | up | histone H1-3 |
| 39 | AT2G18180 | 1.67E-11 | 3.49474464 | up | Sec14p-like phosphatidylinositol transfer family protein |
| 40 | AT2G18193 | 1.18E-08 | 2.32494863 | up | P-loop containing nucleoside triphosphate hydrolases superfamily protein |
| 41 | AT2G20560 | 2.10E-07 | 1.04841164 | up | DNAJ heat shock family protein |
| 42 | AT2G25625 | 1.44E-09 | 1.45883238 | up | uncharacterized protein |
| 43 | AT2G26020 | 7.72E-06 | 1.17082075 | up | putative defensin-like protein |
| 44 | AT2G26650 | 7.20E-08 | 1.01292213 | up | potassium channel AKT1 |
| 45 | AT2G28400 | 0.0001798 | 1.43733158 | up | uncharacterized protein |
| 46 | AT2G29350 | 3.21E-10 | 1.03918036 | up | senescence-associated protein 13 |
| 47 | AT2G33380 | 2.85E-34 | 1.3390005 | up | caleosin 3 |
| 48 | AT2G39330 | 6.35E-72 | 1.17914287 | up | jacalin-related lectin 23 |
| 49 | AT2G41240 | 1.58E-20 | 4.63516879 | up | transcription factor bHLH100 |
| 50 | AT2G41850 | 1.50E-14 | 6.30654602 | up | polygalacturonase ADPG2 |
| 51 | AT2G43580 | 0.0010391 | 3.37226733 | up | chitinase family protein |
| 52 | AT2G46270 | 5.68E-10 | 1.01881805 | up | G-box binding factor 3 |
| 53 | AT2G46680 | 0.00157767 | 1.17991055 | up | homeobox-leucine zipper protein ATHB-7 |
| 54 | AT2G46690 | 1.30E-92 | -8.6594844 | down | SAUR-like auxin-responsive protein |
| 55 | AT3G01840 | 0.00470806 | 2.15498109 | up | LysM-containing receptor-like kinase |
| 56 | AT3G04720 | 1.13E-79 | 1.00638516 | up | pathogenesis-related 4 |
| 57 | AT3G04980 | 0.00559983 | Inf | up | DNAJ heat shock N-terminal domain-containing protein |
| 58 | AT3G08860 | 5.37E-05 | 1.18970959 | up | PYRIMIDINE 4 |
| 59 | AT3G09040 | 4.24E-08 | #NAME? | down | pentatricopeptide repeat-containing protein |
| 60 | AT3G12500 | 1.67E-11 | 1.26741882 | up | basic chitinase B |
| 61 | AT3G12580 | 3.67E-52 | 1.20126283 | up | heat shock protein 70-4 |
| 62 | AT3G19550 | 0.00449261 | 1.43485908 | up | uncharacterized protein |
| 63 | AT3G22840 | 8.64E-07 | -1.1429787 | down | chlorophyll A-B binding, early light-inducible protein |
| 64 | AT3G25780 | 0.00187644 | 1.54725116 | up | allene oxide cyclase 3 |
| 65 | AT3G27250 | 3.25E-05 | 1.03431216 | up | uncharacterized protein |
| 66 | AT3G28740 | 1.17E-05 | -1.0182159 | down | cytochrome P450 CYP81D11 |
| 67 | AT3G29575 | 0.00041514 | 1.05856805 | up | Ninja-family protein AFP3 |
| 68 | AT3G43190 | 0.00682241 | 1.72717854 | up | sucrose synthase 4 |
| 69 | AT3G44300 | 7.63E-08 | 1.20746353 | up | nitrilase 2 |
| 70 | AT3G47340 | 6.91E-19 | 1.04719705 | up | asparagine synthetase [glutamine-hydrolyzing] |
| 71 | AT3G50560 | 6.07E-13 | -1.1698104 | down | Rossmann-fold NAD(P)-binding domain-containing protein |
| 72 | AT3G55240 | 3.29E-06 | 1.24549764 | up | uncharacterized protein |
| 73 | AT3G56970 | 1.02E-15 | 3.27986987 | up | transcription factor ORG2 |
| 74 | AT3G56980 | 0.00069702 | 3.37786056 | up | transcription factor ORG3 |
| 75 | AT3G60140 | 0.0022359 | 1.41179155 | up | beta-glucosidase 30 |
| 76 | AT3G62930 | 0.00167083 | -1.6750543 | down | monothiol glutaredoxin-S6 |
| 77 | AT4G01380 | 2.80E-09 | 1.28570217 | up | plastocyanin-like domain-containing protein |
| 78 | AT4G11650 | 6.77E-14 | 2.50214142 | up | osmotin-like protein OSM34 |
| 79 | AT4G11910 | 0.00012393 | 2.27598282 | up | protein STAY-GREEN2 |
| 80 | AT4G12490 | 3.89E-07 | -1.1619152 | down | Bifunctional inhibitor/lipid-transfer protein/seed storage 2S albumin superfamily protein |
| 81 | AT4G12735 | 2.72E-05 | Inf | up | uncharacterized protein |
| 82 | AT4G14020 | 6.84E-12 | 1.52194098 | up | Rapid alkalinization factor (RALF) family protein |
| 83 | AT4G14080 | 1.08E-05 | Inf | up | putative glucan endo-1,3-beta-glucosidase A6 |
| 84 | AT4G14130 | 0.00089776 | 1.08608083 | up | probable xyloglucan endotransglucosylase/hydrolase protein 15 |
| 85 | AT4G14690 | 0.00197611 | -1.3250301 | down | early light-inducible protein 2 |
| 86 | AT4G16260 | 2.36E-17 | 1.08883688 | up | putative beta-1,3-endoglucanase |
| 87 | AT4G21870 | 2.42E-05 | -1.3281689 | down | heat shock protein class V 15.4 |
| 88 | AT4G21930 | 0.00174725 | 1.0385242 | up | uncharacterized protein |
| 89 | AT4G24000 | 0.00955548 | 1.64667281 | up | cellulose synthase-like protein G2 |
| 90 | AT4G25200 | 3.13E-10 | Inf | up | small heat shock protein 23.6 |
| 91 | AT4G30460 | 2.83E-06 | 1.20941907 | up | glycine-rich protein |
| 92 | AT4G31330 | 0.00417085 | 1.05848357 | up | uncharacterized protein |
| 93 | AT4G32810 | 3.39E-05 | 2.00298064 | up | carotenoid cleavage dioxygenase 8 |
| 94 | AT4G33070 | 0.00043466 | 2.17277094 | up | pyruvate decarboxylase 1 |
| 95 | AT4G34580 | 0.00018075 | 1.03988055 | up | phosphatidylinositol transfer protein COW1 |
| 96 | AT4G37370 | 2.41E-07 | 1.1041782 | up | cytochrome P450, family 81, subfamily D, polypeptide 8 |
| 97 | AT5G01040 | 0.00016918 | -2.4031784 | down | laccase 8 |
| 98 | AT5G01520 | 7.95E-05 | 1.22182509 | up | C3HC4 type RING finger protein |
| 99 | AT5G03210 | 1.53E-06 | 1.82389985 | up | uncharacterized protein |
| 100 | AT5G06510 | 0.00632989 | 2.32476509 | up | nuclear transcription factor Y subunit A-10 |
| 101 | AT5G13210 | 7.80E-09 | 1.82056871 | up | uncharacterized protein |
| 102 | AT5G14120 | 2.00E-162 | -1.2659536 | down | major facilitator protein |
| 103 | AT5G15500 | 1.92E-10 | 1.66849365 | up | ankyrin repeat-containing protein |
| 104 | AT5G17860 | 0.00290769 | 1.20695022 | up | calcium exchanger 7 |
| 105 | AT5G24380 | 3.86E-22 | 1.00294622 | up | metal-nicotianamine transporter YSL2 |
| 106 | AT5G39520 | 5.34E-08 | 3.42915879 | up | uncharacterized protein |
| 107 | AT5G42800 | 2.19E-18 | 1.07154829 | up | dihydroflavonol-4-reductase |
| 108 | AT5G44420 | 1.38E-57 | 1.52194339 | up | ethylene- and jasmonate-responsive plant defensin |
| 109 | AT5G44430 | 5.21E-45 | 1.928972 | up | defensin-like protein |
| 110 | AT5G47590 | 0.00010644 | 1.31278098 | up | Heat shock protein HSP20/alpha crystallin family protein |
| 111 | AT5G50800 | 1.33E-05 | 2.02085587 | up | bidirectional sugar transporter SWEET13 |
| 112 | AT5G53450 | 8.40E-83 | 1.13605586 | up | OBP3-responsive protein 1 |
| 113 | AT5G53820 | 9.70E-06 | 3.52640433 | up | Late embryogenesis abundant protein (LEA) family protein |
| 114 | AT5G53870 | 0.00631205 | 1.16048244 | up | early nodulin-like protein 1 |
| 115 | AT5G54190 | 2.39E-05 | 1.00294283 | up | protochlorophyllide reductase A |
| 116 | AT5G59220 | 7.98E-14 | 1.3823053 | up | putative protein phosphatase 2C 78 |
| 117 | AT5G59305 | 0.00016664 | 2.32481898 | up | uncharacterized protein |
| 118 | AT5G62730 | 0.00482883 | -1.6070091 | down | probable peptide/nitrate transporter |
| 119 | AT5G62920 | 3.52E-15 | -1.1709717 | down | two-component response regulator ARR6 |
| 120 | Arab_newGene_186 | 8.01E-05 | -1.7697577 | down | peptide chain release factor, putative; 62597-62246 |
| 121 | Arab_newGene_295 | 1.74E-05 | Inf | up | - |
| 122 | Arab_newGene_36 | 0.00576104 | 2.63514468 | up | - |
| 123 | Arab_newGene_48 | 0.00234404 | 1.12497349 | up | - |
| 124 | Arab_newGene_85 | 5.94E-06 | -3.697157 | down | - |

Note: “up” indicate upregulation of gene and “down” indicate downregulation of gene
